# SNORD6 promotes cervical cancer progression by accelerating E6-mediated p53 degradation

**DOI:** 10.1038/s41420-023-01488-w

**Published:** 2023-06-27

**Authors:** Qianhui Li, Bumin Xie, Xi Chen, Bingfeng Lu, Shuo Chen, Xiujie Sheng, Yang Zhao

**Affiliations:** Department of Obstetrics and Gynecology, Department of Gynecologic Oncology Research Office, Guangzhou Key Laboratory of Targeted Therapy for Gynecologic Oncology, Guangdong Provincial Key Laboratory of Major Obstetric Diseases, The Third Affiliated Hospital of Guangzhou Medical University, Guangzhou, 510150 China

**Keywords:** Oncogenes, Oncogenesis

## Abstract

Small nucleolar RNAs (snoRNAs) are a class of non-coding RNAs widely distributed in eukaryotic nucleoli. In recent years, studies have revealed that snoRNAs can also participate in the occurrence and development of malignant tumors through different pathways. Cervical cancer is one of the most common malignant tumors of the female reproductive system, and the high-risk HPV virus infection is its main pathogenic mechanism. However, the outcomes in different patients with malignant tumors vary, indicating that other factors might affect the pathogenic process of cervical cancer. In this study, we screened the poor prognosis indicator SNORD6 from the TCGA database to find the snoRNA that affects the disease outcome during the pathogenesis of cervical cancer. We discovered that SNORD6 expression in cervical cancer tissues was higher than that in normal cervical tissues. Cell phenotype experiments revealed that the knockdown of SNORD6 retarded cell proliferation and plate clone formation. Furthermore, G1-S phase cell cycle arrest was induced, DNA synthesis was decreased, cell migration and invasion were reduced, while the level of apoptosis increased, whereas the opposite results were obtained after SNORD6 overexpression. Moreover, after intratumoral injection of ASO-SNORD6, the tumor growth rate slowed down, and the tumor volume decreased compared with the control group. In the mechanism study, we found that SNORD6 concurrently acted as a binding “hub” to promote the formation of the tumor suppressor protein p53 degradation complex E6-E6AP-p53. This reaction enhanced the ubiquitination and degradation of p53, thus influenced the regulation of p53 activities in the cell cycle and apoptosis. This study preliminarily clarified the biological role and specific mechanism of SNORD6 in the occurrence of cervical cancer, broadening the basic theoretical research of ovarian cancer and may provide a new perspective on the diagnosis and treatment of cervical cancer.

## Introduction

Cervical cancer is the fourth most common malignancy and the fourth leading cause of cancer death in women worldwide, with approximately 604,000 new cases and 342,000 deaths in 2020 worldwide [1]. Human papillomavirus (HPV) is the most prominent risk factor for cervical carcinogenesis, and more than 90% of cervical cancers are accompanied by high-risk HPV infection, whose types 16 and 18 are most closely associated with cervical carcinogenesis [2–5]. In addition, the risk of HPV infection is increased in immunocompromised populations, in those with multiple sexual partners and a first sexual encounter <16 years, and in women with a first birth at a younger age. Cervical cancer is mainly treated with surgery and radiotherapy-based, chemotherapy adjuvant comprehensive treatment regimens.

HPV is a virus with a non-enveloped double-stranded DNA and epitheliotropic properties, composed of nucleic acid and capsid protein. Its genome can be divided into an early coding region (E region), late coding region (L region), and long control region (LCR) [6–8]. More than 200 types of HPV viruses have been isolated, classified into high-risk and low-risk HPV viruses based on their pathogenicity and carcinogenesis potential [7, 9]. Earlier studies have shown that the persistent infection of high-risk HPV is closely related to the occurrence of high-grade cervical lesions (HSIL) and cervical cancer [10, 11]. When the HPV virus infects the organism, its genome can randomly integrate into the host cell DNA. In such a way, it subsequently silences the expression of the E2 gene, weakens the inhibition of the E6 or E7 gene by the E2 protein, and causes E6 or E7 protein overexpression. Then, it promotes the immortalization or even the carcinogenesis of the infected cell. However, the post-carcinogenesis outcomes in different patients are not the same. Hence, we considered that other factors might play a supplementary regulatory role in the pathogenic process of cervical cancer.

Small nucleolar RNAs (snoRNAs) are a class of small non-coding RNAs with an approximate length of 60–300 nucleotides [12]. snoRNAs are divided into three categories according to their conserved structural elements: box C/D snoRNA, box H/ACA snoRNA, and small Cajal-body specific ribonucleic acid (scaRNA) [13–15]. Of them, box C/D and box H/ACA are the main types of known snoRNAs, which guide the 2 ´-O methylation and pseudouridylation of ribosomal RNA (rRNA) in a base-pairing manner, respectively. Studies have found that in addition to modifying rRNA, snoRNA can also modify other RNAs, such as snRNA, mRNA, and tRNA [16–19]. Additionally, it can affect biological processes such as ribosome biosynthesis and alternative splicing. Recent research established that snoRNA is involved in the occurrence and development of various malignant tumors through many different mechanisms. For example, SNORD50A and SNORD50B which are repeatedly deleted in human tumors are directly bound to and inhibit K-Ras. Meanwhile, the deletion of SNORD50A and SNORD50B enhanced K-Ras mutation-induced tumorigenesis [20]. Moreover, SNORA18L5 promotes the progression of HBV-related hepatocellular carcinoma by regulating the nucleocytoplasmic translocation of ribosomal proteins RPL5 and RPL11 and the degradation level of p53 [21]. snoRNAs (SNORA73A, SNORA73B, and SNORA74A) can promote ribosome formation and breast cancer cell growth by binding to PARP-1 and promoting its ADP ribosylation of DDX21 [22]. However, the participation of snoRNAs in the pathogenic mechanisms of the occurrence and development of cervical cancer has not yet been reported. In this study, we screened the TCGA database to determine the snoRNAs that affect the disease outcome during the occurrence and development of cervical cancer. We established that SNORD6 was associated with poor prognosis in cervical cancer. Histological examination showed that the expression level of SNORD6 in cervical cancer tissues was higher than that in normal cervical epithelial tissues, suggesting that SNORD6 may play a tumor-promoting role in cervical cancer. Therefore, here, we selected SNORD6 as a research object and explored its role and detailed mechanism in the occurrence and development of cervical cancer.

## Results

### SNORD6 is associated with poor cervical cancer prognosis

**Fig. 1:**
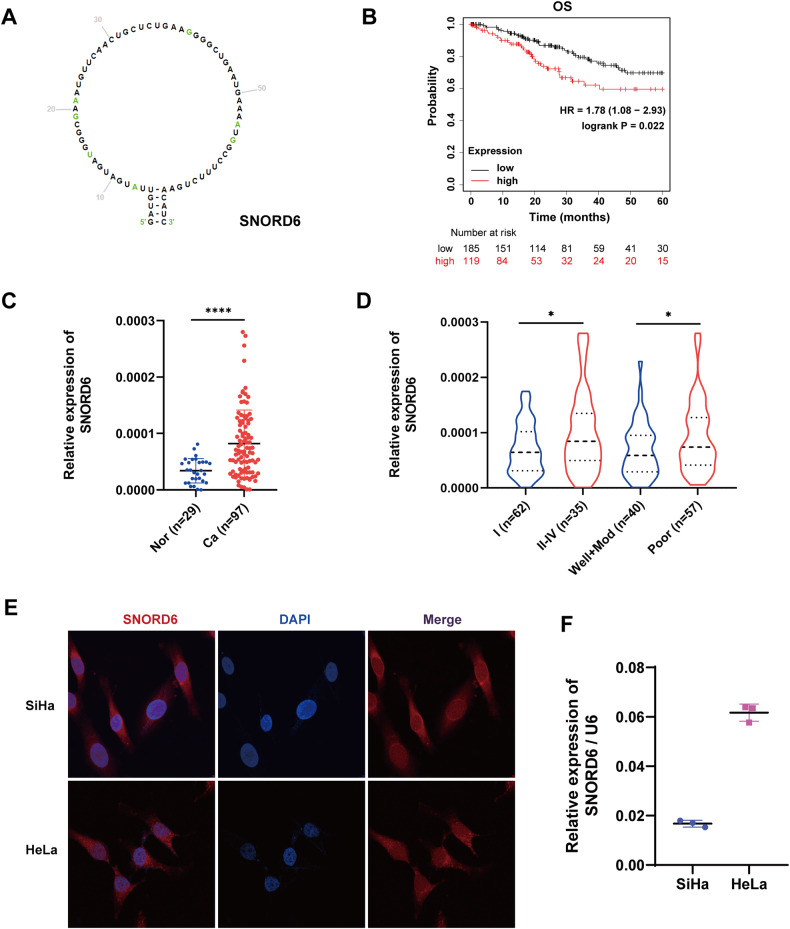
SNORD6 is associated with poor prognosis in cervical cancer. **A** Diagram showing the structure of SNORD6. **B** Kaplan–Meier analysis of the correlation between SNORD6 expression and the 5-year OS in cervical cancer patients. **C** qRT-PCR results of SNORD6 expression in cervical cancer tissue and normal cervical epithelial tissue. **D** The expression level of SNORD6 in cervical cancer tissue of FIGO stage II–IV is higher than that of cervical cancer tissue of FIGO stage I (left). The expression level of SNORD6 in poorly differentiated cervical cancer tissues is higher than that in moderately and well differentiated cervical cancer tissues (right). **E** The localization of SNORD6 in SiHa and HeLa cell line was detected by FISH experiment. Scale bar, 20 μm. Red: SNORD6; blue: DAPI. **F** The expression of SNORD6 in cervical cancer cell lines SiHa and HeLa was detected by qRT-PCR. Datas are expressed as the mean ± SD of the (**C&F**) graph; (**D**) graphs are expressed as the median (Q1, Q3). **C**, **D,** **F**, Student’s *t* test. *p < 0.05, ****p < 0.0001.

In this study, we screened the TCGA database (https://portal.gdc.cancer.gov/) and found that the prognosis of cervical cancer patients with high expression of the snoRNA SNORD6 (Fig. 1A) was worse than that of patients with low expression. The 5-year overall survival (OS) (Fig. 1B) of patients were statistically significant (http://kmplot.com/analysis/) [23]. Then, the expression level of SNORD6 was detected in 29 normal cervical epithelial tissues and 97 cervical cancer tissues. The results showed that the expression of SNORD6 in the cervical cancer tissues was higher than that in the normal cervical epithelial tissues (Fig. 1C) (Supplementary Table 1). In addition, the expression of SNORD6 in the cervical cancer tissues in the FIGO stage II–IV was higher than that in stage I (Fig. 1D left). Its expression in poorly differentiated cervical cancer tissues was higher than that in moderately and well-differentiated cervical cancer tissues (Fig. 1D right) (Supplementary Table 2). This suggests that SNORD6 may play a role in cervical carcinogenesis.

### SNORD6 plays a cancer-promoting role in cervical cancer cells

FISH experiments were performed to determine the localization of SNORD6 in the cervical cancer cells. The results showed that SNORD6 was expressed in both the cytoplasm and the nucleus (Fig. 1E). Then, we detected the expression of SNORD6 in the cervical cancer cell lines SiHa and HeLa, and found that the expression level of SNORD6 in the HeLa cells was higher than that in the SiHa (Fig. 1F). Therefore, we performed SNORD6 expression knockdown experiments in the HeLa cell line and SNORD6 overexpression experiments in the SiHa cell line.

**Fig. 2:**
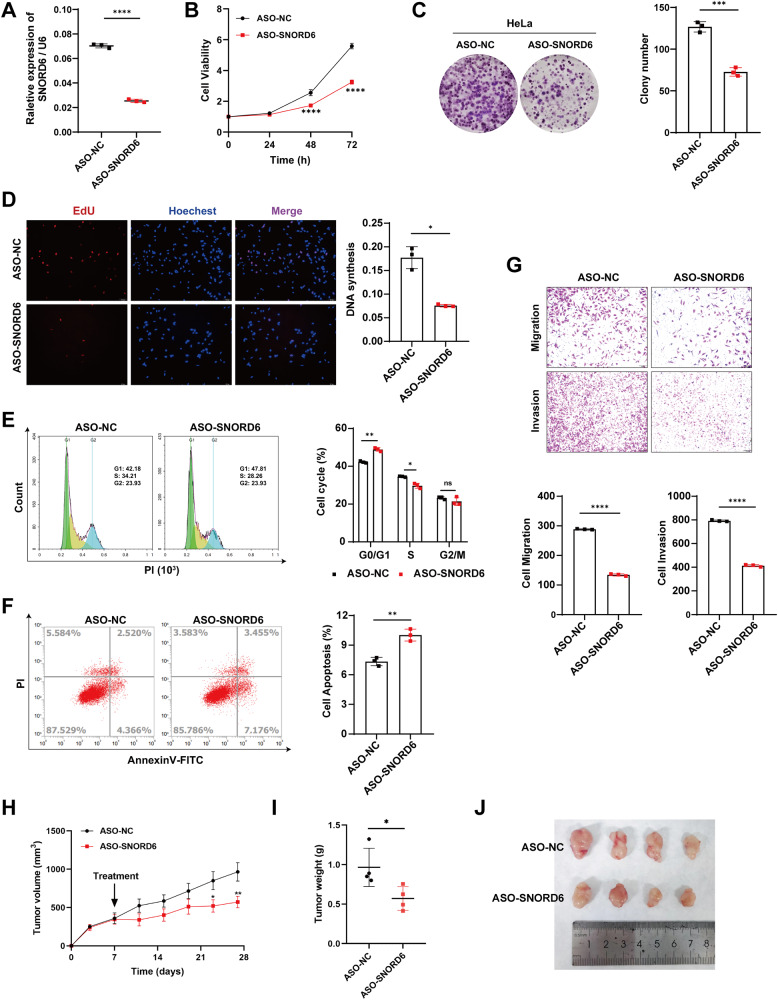
After knockdown of SNORD6, cell proliferation, migration and invasion slowed down, and apoptosis increased. **A** The expression level of SNORD6 decreased after transfection of ASO-SNORD6 in HeLa cells. Knockdown of SNORD6 in HeLa cells resulted in decreased cell proliferation rate (**B**), decreased colony formation (**C**), slowed DNA synthesis (**D**), arrested cell cycle progression (**E**), and increased apoptosis (**F**), decreased migration and invasion (**G**). **H** After ASO-SNORD6 treatment, the tumor growth rate slowed down compared to the control group. **I** The total tumor mass of ASO-SNORD6 treatment group was smaller than that of the control group. **J** The tumor volume of the ASO-SNORD6 treatment group was smaller than that of the control group. In (**D**, **G**) scale bar, 100 μm. All experiments were repeated three times independently. The above data are expressed as mean ± SD. Student’s *t* test. *p < 0.05, **p < 0.01, ***p < 0.001, ****p < 0.0001.

The expression of SNORD6 was knocked down by antisense oligonucleotide (ASO) technology. Q-PCR was used to check the transfection efficiency. We established that the expression level of SNORD6 was significantly reduced after the transfection of ASO-SNORD6 in HeLa cells (Fig. 2A). CCK-8 and plate cloning experiments were conducted to detect the changes in the cell proliferation levels. The results showed that the cell proliferation rate was significantly reduced after the SNORD6 knockdown (Fig. 2B); the number of formed cell clones also significantly decreased (Fig. 2C). EdU staining analysis and flow PI staining were next implemented to detect the cell cycle changes. The results of the EdU experiments showed that the number of cells with EdU incorporation in DNA after the SNORD6 knockdown was significantly lower than that in the control group (Fig. 2D). The cell cycle was arrested in the G1-S phase after the SNORD6 knockdown (Fig. 2E). The results of the flow cytometry Annexin V-FITC/PI staining, applied to detect apoptosis, showed that the apoptosis level was increased after the SNORD6 knockdown (Fig. 2F). Transwell assay results showed that the decreased expression of SNORD6 was associated with reduced cell migration and invasion (Fig. 2G). ASO-SNORD6 treatment experiments after tumorigenesis in nude mice mimic the effect of SNORD6 in vivo. Results showed that after intratumoral injection of ASO-SNORD6, the tumor growth rate slowed down (Fig. 2H), and the tumor volume decreased compared with the control group (Fig. 2I, J).

**Fig. 3:**
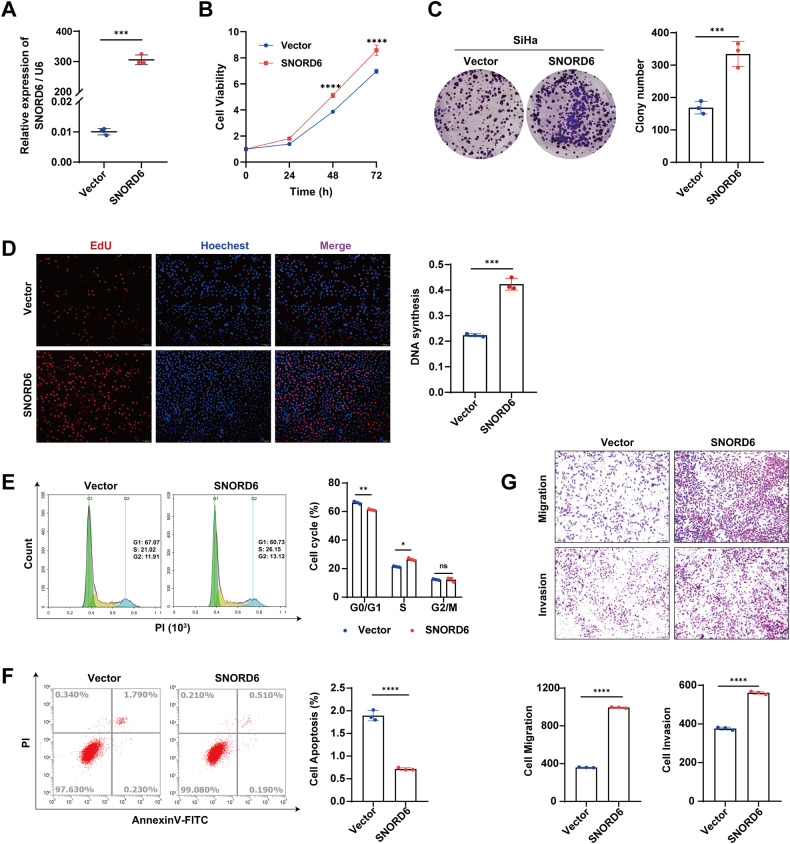
SNORD6 plays a promoting role in cervical cancer cells. **A** Q-PCR results showing the transfection efficiency after transfection of SNORD6 overexpression plasmid in SiHa. Overexpression of SNORD6 in SiHa resulted in increased cell growth (**B**), increased colony formation (**C**), increased DNA synthesis (**D**), increased cell cycle progression (**E**), decreased apoptosis (**F**), increased migration and invasion (**G**). In (**D**, **G**) scale bar, 100 μm. All experiments were repeated three times independently. The above data are expressed as mean ± SD. Student’s *t* test. *p < 0.05, **p < 0.01, ***p < 0.001, ****p < 0.0001.

After the transfection of SNORD6 overexpression plasmids in the SiHa cell line, the SNORD6 expression levels were significantly increased (Fig. 3A). Compared with the empty vector group, the overexpression of SNORD6 increased the rate of cell proliferation (Fig. 3B), increased the formation of plate clones (Fig. 3C), significantly increased the number of cells in S phase stained by EdU (Fig. 3D), accelerated the progression of the G1-S phase of the cell cycle (Fig. 3E), decreased the level of apoptosis (Fig. 3F), and increased cell migration and invasion (Fig. 3G). Another human cervical cancer cell line CaSki showed the same results (Supplementary Fig. 1). The above results indicate that SNORD6 plays a tumor-promoting role in cervical cancer cells.

### SNORD6 can bind to E6 protein

**Fig. 4:**
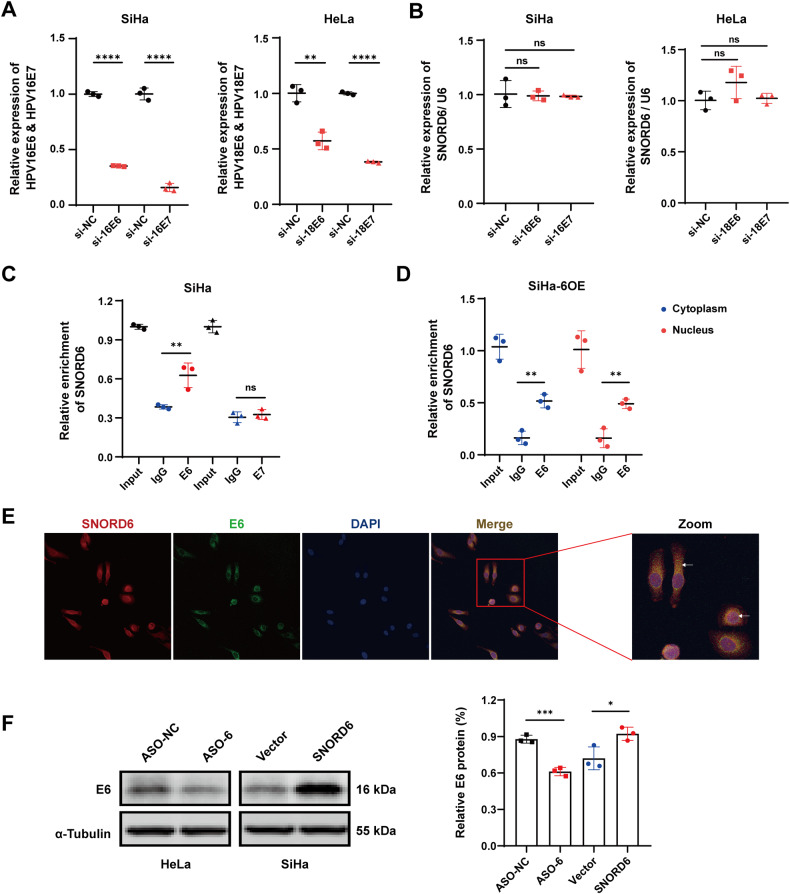
SNORD6 can bind to HPV E6 protein. **A** Knockdown of HPV-16E6/HPV-16E7 in SiHa cells and knockdown of HPV-18E6/HPV-18E7 in HeLa cells mRNA level transfection efficiency. **B** Q-PCR results showing SNORD6 expression level after knockdown of HPV-16E6/HPV-16E7 in SiHa cells and knockdown of HPV-18E6/HPV-18E7 in HeLa cells does not change. **C** RIP results showed that SNORD6 physically bound to HPV E6 protein, but not to HPV E7 protein. **D** SNORD6 in both cytoplasm and nucleus can bind to E6 in SiHa cells overexpressing SNORD6. SiHa-6OE: SiHa cells overexpressing SNORD6. **E** Immunofluorescence-FISH experiments showed that SNORD6 co-localized with E6 protein. Scale bar, 20 μm. Red: SNORD6; green: E6; blue: DAPI. **F** HPV E6 protein expression increased after SNORD6 overexpression and decreased after SNORD6 knockdown. ASO-6: ASO-SNORD6. All experiments were repeated three times independently. The above data are expressed as mean ± SD. Student’s *t* test. *p < 0.05, **p < 0.01, ***p < 0.001, ****p < 0.0001.

Recent studies have shown that the occurrence of cervical cancer is related mainly to high-risk HPV infection [3, 5, 10, 11]. High-risk HPV viruses can integrate into the host genome, and subsequent overexpression of E6 and E7 oncoproteins leads to infinite proliferation and even canceration of the infected cells. Therefore, in this study, we explored whether the cancer-promoting effect of SNORD6 is related to the expression of E6 and E7 proteins. The expression of E6 and E7 proteins was silenced by transfecting small interfering RNA (siRNA) in cells, and the transfection efficiency was detected by Q-PCR (Fig. 4A). The results showed that the expression level of SNORD6 did not change significantly after siE6/E7 (Fig. 4B). This suggests that E6 and E7 proteins may not exert a regulatory effect on SNORD6 expression.

Then, we aimed to determine whether SNORD6 could play a role by binding to E6 and E7 proteins. RIP experiment results revealed that SNORD6 could bind to E6 protein, but not to E7 protein (Fig. 4C). In addition, SNORD6 in both cytoplasm and nucleus can bind to E6 in SiHa cells overexpressing SNORD6 (Fig. 4D). Consistent with this result, the immunofluorescence-FISH experiments showed that SNORD6 was co-localized with E6 proteins (Fig. 4E). Therefore, SNORD6 may play a role in promoting cancer by binding to E6 protein. Next, we analyzed whether this binding could affect E6 protein expression by detecting the changes in the E6 protein expression after SNORD6 overexpression or knockdown. SNORD6 overexpression significantly increased the E6 protein levels, whereas they were significantly decreased after the knockdown of SNORD6 (Fig. 4F). Another cervical cancer cell line CaSki showed the same results (Supplementary Fig. 2A).

### SNORD6 plays a “hub” role in the formation of the p53 degradation complex

**Fig. 5:**
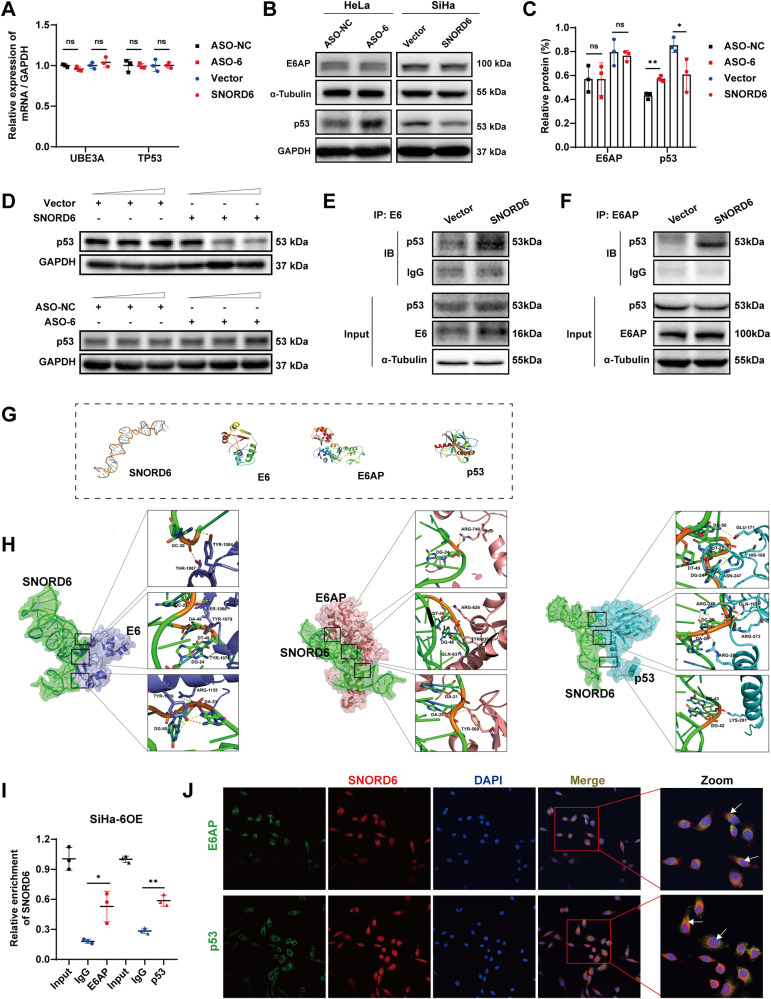
SNORD6 promotes the formation of E6/E6AP/p53 degradation complex. **A** The mRNA expression of UBE3A (E6AP) and TP53 did not change after the SNORD6 overexpression and knockdown. **B**, **C** The protein expression of E6AP did not change after the SNORD6 overexpression and knockdown. The protein expression of p53 was decreased after overexpression of SNORD6, and the expression of p53 protein was increased after knockdown of SNORD6. **D** The effect of SNORD6 on p53 protein expression was dose-dependent. The concentrations of Vector and SNORD6 were: 0μg, 5μg, 10μg. The concentrations of ASO-NC and ASO-SNORD6 were: 0nM, 50nM, 100nM. Co-IP results show enhanced endogenous binding between E6-p53 (**E**) and E6AP-p53 (**F**) after SNORD6 overexpression. **G** The tertiary structure of SNORD6 and protein 3D structure of E6, E6AP and p53 in the PDB database. **H** The result of molecular docking between SNORD6 and E6, E6AP and p53. **I** RIP-qPCR results showed that SNORD6 was physically bound to both E6AP and p53. **J** FISH-immunofluorescence results showed that SNORD6 co-localized with E6AP and p53. Scale bar, 20 μm. Red: SNORD6; green: E6AP or p53; blue: DAPI. ASO-6: ASO-SNORD6; SiHa-6OE: SiHa cells overexpressing SNORD6. All experiments were repeated three times independently. The above data are expressed as mean ± SD. Student’s *t* test. *p < 0.05, **p < 0.01.

Studies have shown that E6 protein binds mainly to the p53 protein to promote p53 degradation [24–27]. Under normal circumstances, E6 protein first binds to E3 ubiquitin ligase E6AP to form a complex E6/E6AP, and then binds to p53. E6AP transfers the activated ubiquitin to p53 protein, which is degraded by the proteasome. Therefore, we analyzed whether the binding of SNORD6 to E6 protein can affect the above process. First, this study analyzed the changes in the expression levels of E6AP and p53 after SNORD6 overexpression or knockdown. The results showed that mRNA of E6AP and p53 did not change (Fig. 5A) while only protein levels of p53 changed, the p53 protein expression level was significantly decreased after SNORD6 overexpression and significantly increased after SNORD6 knockdown (Fig. 5B, C). Another cervical cancer cell line CaSki showed the same results (Supplementary Fig. 2A). Moreover, the effect of SNORD6 on p53 protein expression was dose-dependent (Fig. 5D). These results suggest that SNORD6 can also influence the expression of the p53 protein. However, it still remains unclear how SNORD6 induces changes in p53 protein expression.

In this study, the changes in the binding capacity of E6-p53 and E6AP-p53 were detected after the in vitro overexpression of SNORD6. Co-IP results revealed that the endogenous covalent binding between E6-p53 (Fig. 5E) and E6AP-p53 (Fig. 5F) was enhanced after SNORD6 overexpression. This study then analyzed why SNORD6 enhanced the binding ability between the three proteins. The 3dRNA tool was used to predict the tertiary structure of SNORD6 (Fig. 5G). After obtaining the protein 3D structure of E6, E6AP, and p53 in the PDB database (Fig. 5G), the RNA-protein molecular docking prediction was performed using HDOCK. The results showed that SNORD6 may interact well with E6, E6AP, and p53 (Fig. 5H). In result 3, the physical binding of SNORD6 to E6 was confirmed, and then we used RIP-qPCR experiments to verify the binding effect of SNORD6 to E6AP and p53. We found that SNORD6 could indeed bind to E6AP and p53 (Fig. 5I). Meanwhile, immunofluorescence-FISH results showed that SNORD6 was co-localized with E6AP and p53 (Fig. 5J). As it can be seen, SNORD6 plays a “hub” role in the formation of the p53 degradation complex E6/E6AP/p53 and promotes the formation of the E6/E6AP/p53 degradation complex.

### SNORD6 promotes the ubiquitination and degradation of p53

**Fig. 6:**
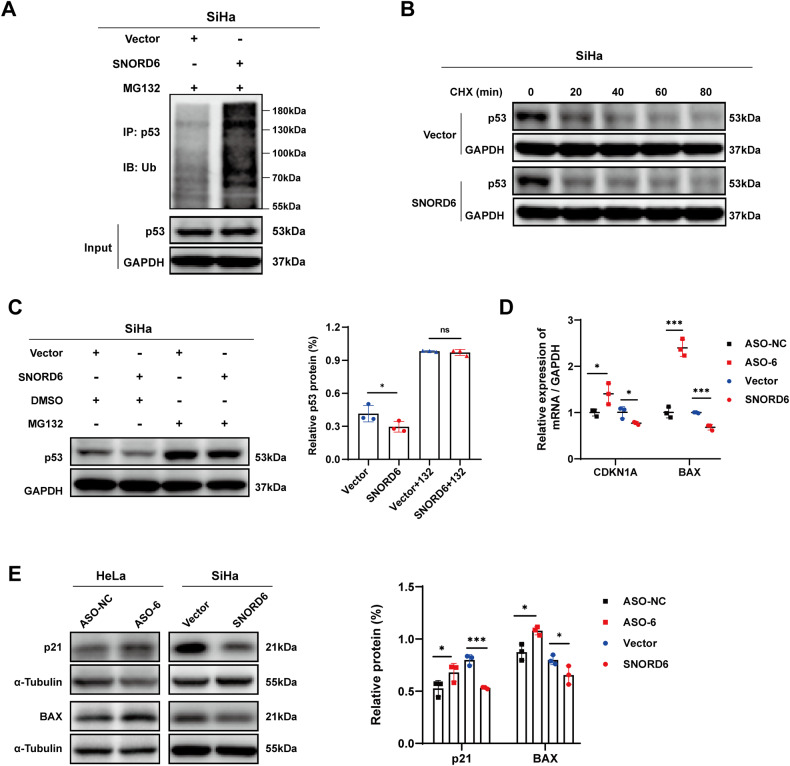
SNORD6 promotes ubiquitination and degradation of p53. **A** Western blot results showed that the level of p53 ubiquitination was significantly increased after SNORD6 overexpression. **B** After cycloheximide (CHX) (80 μg/ml) treatment, the degradation rate of p53 protein in the SNORD6 overexpression group was accelerated. **C** The accelerated degradation of p53 protein caused by SNORD6 overexpression group was prevented after MG132 (20 μM) treatment. **D** Q-PCR results showed that the mRNA expression of p53 downstream genes CDKN1A and BAX decreased after SNORD6 overexpression, and the opposite results after SNORD6 expression knockdown. **E** Western blotting results showed that the expression of p53 downstream proteins p21 and BAX decreased after SNORD6 overexpression, and the opposite results after SNORD6 expression knockdown. ASO-6: ASO-SNORD6. All experiments were repeated three times independently. The above data are expressed as mean ± SD. Student’s *t* test. *p < 0.05, ***p < 0.001.

Since E6/E6AP is involved mainly in the ubiquitin-proteasomal degradation of p53, we analyzed the effect of SNORD6 on the level of p53 ubiquitination. The results showed that the level of p53 ubiquitination was significantly increased after SNORD6 overexpression (Fig. 6A). This result suggests that SNORD6 promotes the ubiquitination degradation of p53. In addition, the protein synthesis inhibitor cycloheximide (CHX) was used for treatments of the SNORD6 overexpression and the control group, respectively. Subsequently, we established that the degradation rate of the p53 protein was accelerated after SNORD6 overexpression (Fig. 6B). Meanwhile, the treatment with the proteasome inhibitor MG132 returned to the baseline level of the p53 protein content in the SNORD6 overexpression and the control groups (Fig. 6C). The aforementioned results confirm that SNORD6 affects the ubiquitin-proteasome degradation process of the p53 protein.

Several studies have confirmed that p53 plays an important role in the regulation of the cell cycle and apoptosis [28–31]. Hence, we further assessed the potential effects of SNORD6 on p53-mediated cell cycle and apoptosis. In previous results (Fig. 3E, F), it became clear that SNORD6 overexpression promoted the G1-S phase progression of the cell cycle and inhibited the apoptosis process. Subsequent studies found that the expression of apoptosis-related gene BAX downstream of p53 and the cell cycle regulatory gene CDKN1A decreased after the overexpression of SNORD6, whereas the opposite results were obtained after the knockdown of SNORD6 expression (Fig. 6D, E). The same results can be observed in CaSki (Supplementary Fig. 2B, C). In conclusion, SNORD6 influences the process of cervical carcinogenesis by regulating the E6/E6AP-mediated ubiquitination and degradation of p53, thereby affecting the regulatory effect of p53 on the cell cycle and apoptosis.

### The cancer-promoting effect of SNORD6 is p53-dependent

**Fig. 7:**
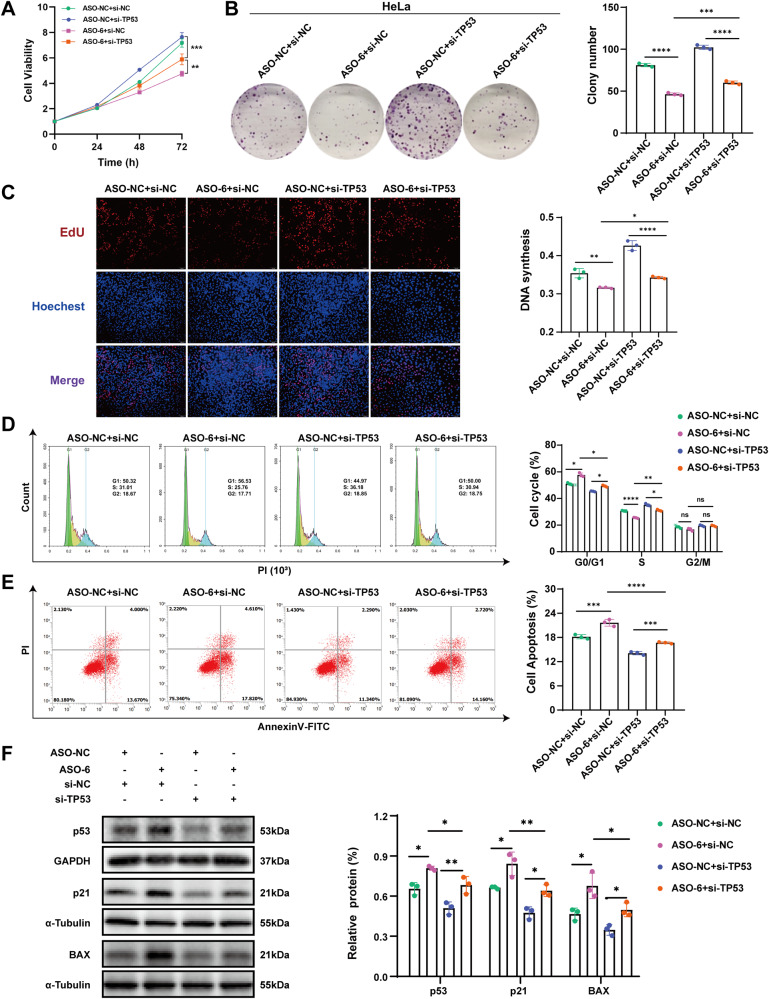
The tumor-promoting effect of SNORD6 depends on p53. Altered cell proliferation rate (**A**), plate colony formation (**B**), DNA replication levels (**C**), cell cycle progression (**D**), cell apoptosis (**E**) and changes in genes downstream of p53 (**F**) could be recovered. In (**C**) scale bar, 100 μm. ASO-6: ASO-SNORD6. All experiments were repeated three times independently. The above data are expressed as mean ± SD. One-way ANOVA. *p < 0.05, **p < 0.01, ***p < 0.001, ****p < 0.0001.

The aforementioned results have elucidated that SNORD6 can promote the formation of a p53 ubiquitination and degradation complex (E6/E6AP/P53) by acting as a “hub”. Hence, it promotes the process of p53 ubiquitination and degradation, affecting its regulatory activity in cell cycle and apoptosis. Therefore, in the present study, we next explored whether the tumor-promoting effect of SNORD6 was dependent on its regulation of p53 expression. After the knockdown of SNORD6 expression, TP53 small interfering RNA (si-TP53) was co-transfected to detect the recovery of si-TP53 to the cell phenotype and the expression of p53 downstream proteins after the SNORD6 knockdown. The results showed that after knockdown SNORD6 and co-transfected with si-TP53, the cell proliferation rate (Fig. 7A), plate colony formation (Fig. 7B), DNA synthesis (Fig. 7C), cell cycle progression (Fig. 7D), and cell apoptosis (Fig. 7E) were all recovered. Meanwhile, the expression levels of the p53 protein and its downstream p21 and BAX were decreased (Fig. 7F). In addition, we next co-transfected the TP53 overexpression plasmid and the empty plasmid with ASO-NC or ASO-SNORD6, respectively. The results indicated that overexpression of TP53 further enhanced the slowing down of cell proliferation and increased apoptosis caused by SNORD6 knockdown, p53 and its downstream target proteins also showed similar changes (Supplementary Fig. 3). It can be seen that the tumor-promoting effect of SNORD6 in cervical cancer cells is p53-dependent.

## Discussion

Cervical cancer is one of the most common malignant tumors of the female reproductive system. In the 1980s, Harold Zur Hausen first proposed that HPV infection is an important carcinogen of cervical cancer [10, 11]. Further studies have since confirmed that approximately 98.5% of invasive cervical cancers are associated with HPV infection [2]. After HPV infects the body, the probability of cancer development and the length of survival after cancer are quite different among patients, which suggests that regulatory factors other than the HPV virus may be involved in the pathogenesis of cervical cancer. snoRNAs are a class of non-coding RNAs that play an important regulatory role in eukaryotic ribosome biogenesis and post-transcriptional modification of different RNA types. In recent years, studies have gradually elucidated that snoRNAs has an important regulatory role in the occurrence and development of malignant tumors [32–36]. In this study, to establish the snoRNAs that affect the prognosis of cervical cancer, we screened the poor prognosis indicator in the TCGA database. Histological examinations found that the SNORD6 expression levels in cervical cancer tissues were significantly higher than those in normal cervical epithelial tissues. SNORD6 expression was positively correlated with tumor FIGO stage and differentiation. Cytological assessment showed that after SNORD6 knockdown, the growth rate of cervical cancer cells was retarded, the formation of plate clones was reduced, the level of apoptosis was increased, the G1-S phase of the cell cycle was blocked, and the number of cells with EdU incorporation in DNA was significantly decreased, cell migration and invasion were reduced. These effects were reversed after SNORD6 overexpression. In vivo tumorigenesis assay in nude mice showed that after ASO-SNORD6 treatment, the tumor growth rate was slowed and the tumor size was smaller compared with the control group. Therefore, SNORD6 plays a promoting role in cervical carcinogenesis.

After infections with high-risk HPV, its DNA usually integrates into the host cell genome, resulting in deletion or mutation of host genes. As a result, the expression of oncogenes and tumor suppressor genes in the host cells is affected. The E6 and E7 proteins are the major oncogenic proteins expressed by the HPV genome. Therefore, this study explored whether E6 and E7 are involved in the oncogenic role of SNORD6 in cervical cancer. The results showed that silencing of E6 and E7 failed to change the expression of SNORD6, suggesting that SNORD6 was not regulated by E6 and E7. We then intended to find out whether SNORD6 exerted a regulatory role by binding to the E6 or E7 proteins. The RIP-qPCR results confirmed that there was a physical association between SNORD6 and E6, but not E7. Immunofluorescence-FISH experiments verified the co-localization of SNORD6 and E6 proteins. Hence, we needed to understand whether this binding can affect the function of the E6 protein. We found that E6 protein expression could be increased or decreased by SNORD6-overexpression or silencing.

The E6 protein is involved in the induction of cervical carcinogenesis via many pathways, and the interaction between the E6 and p53 proteins is essential in cervical carcinogenesis [24]. It is generally believed that E6 protein can prevent the normal function of the p53 protein by ubiquitinating it through the E6-associated protein (E6-AP) and decomposing it [25]. E6AP, a member of the HECT E3 ubiquitin ligase family, was originally identified as an interacting protein of the E6 oncoprotein, and later studies revealed that the E6-E6AP complex was able to target the tumor suppressor p53 (in the absence of E6, E6AP does not target p53), which in turn promoted the ubiquitination and degradation of p53, ultimately leading to HPV-induced cervical carcinogenesis [26, 27]. Therefore, this study explored the effect of SNORD6 on the binding capacity of E6-p53 and E6AP-p53. The results showed that the binding capacity of E6-p53 and E6AP-p53 were enhanced after SNORD6 overexpression. Then, we analyzed the reasons for the above-mentioned enhanced binding effect. Molecular docking results showed that SNORD6 might bind to E6, E6AP, and p53. RIP-qPCR results showed that SNORD6 was physically bound to the three proteins. Immunofluorescence-FISH results evidenced that SNORD6 was co-localized with the three proteins. These results suggested that SNORD6 may play a “hub” linking role in the formation of the p53 ubiquitination degradation complex. Meanwhile, further research found that the ubiquitination modification level of p53 was significantly elevated after SNORD6 overexpression. In addition, the p53 degradation rate was also significantly accelerated after SNORD6 overexpression compared with that of the control group. After adding the proteasome inhibitor MG132, no significant difference in the p53 protein expression between the control group and the SNORD6 overexpression group was observed. Therefore, SNORD6/E6/E6AP was indeed involved in the ubiquitin-proteasome degradation pathway of p53. In conclusion, SNORD6 can act as a junction hub to facilitate the connection of E6/E6AP/p53, thereby promoting the ubiquitination and degradation of p53.

As a tumor suppressor, the p53 protein participates in multiple biological processes in the body’s cells, including cell cycle, DNA damage repair, and apoptosis [28]. When the DNA damage is mild, the expression level of p53 protein in the body increases, so that the damaged cells stay at the G1 checkpoint for DNA repair, thereby maintaining the integrity of the cell genome. When DNA repair fails, the p53 protein initiates the apoptosis pathway, induces cell aging or apoptosis, thereby reducing the probability of genomic mutations [37]. Therefore, the silencing or mutation of the TP53 gene may disturb the intracellular signal transduction pathway, disabling the control of cell growth and apoptosis process and inducing cell canceration [38]. p53 functions predominantly as a transcription factor to activate or suppress a large number of its downstream genes. The CDKN1A gene encodes a cyclin-dependent kinase inhibitor, p21, the first transcriptional target of p53 discovered, which can inhibit cyclin kinases, including CDK1, CDK2, CDK3, CDK4, and CDK6, and mediates p53-induced G1 cell cycle arrest [30, 39–41]. As a pro-apoptotic protein, BAX is a member of the BCL-2 family. It was found that when the DNA-binding element of p53 binds to the BAX promoter region, its expression is up-regulated, that is, BAX can act as a downstream target of p53 [31, 42, 43]. In this study, we found that p53 was downregulated upon SNORD6 overexpression, which in turn caused a decrease in the expression of its downstream target genes CDKN1A and BAX. Opposite results were obtained when SNORD6 was knocked down.

**Fig. 8:**
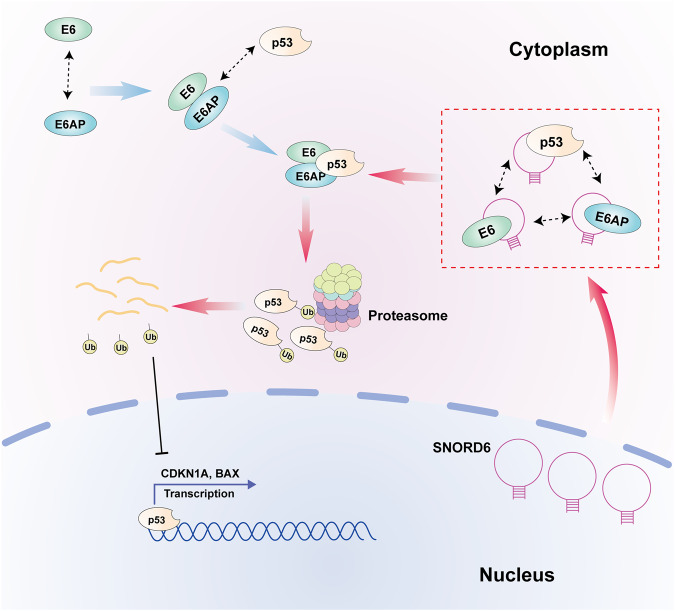
The role of SNORD6 in cervical cancer. SNORD6 acts as a “hub” to promote the formation of the p53 ubiquitination degradation complex E6/ E6AP/ p53 and then facilitates p53 protein degradation, which leads to the abnormal regulation of p53 in the cell cycle and apoptosis.

Then, this study explored whether the tumor-promoting effect of SNORD6 in cervical cancer depends on its regulation of p53 expression. The results showed that after knockdown of SNORD6 and transfection of si-TP53, the slowdown of cell proliferation, clony formation, DNA synthesis, cell cycle arrest and increased cell apoptosis caused by knockdown of SNORD6 could be recovered. At the same time, the elevated expression of p53, p21, and BAX caused by knockdown of SNORD6 could also be restored. This indicates that the tumor-promoting effect of SNORD6 in cervical cancer depends on p53. In conclusion, this study confirms that SNORD6 plays a promoting role in cervical carcinogenesis, which acts as a “hub” to promote the formation of the p53 ubiquitination degradation complex E6/ E6AP/ p53 and then accelerates p53 protein degradation, leading to the abnormal regulation of p53 in the cell cycle and apoptosis, then promotes the development of cervical cancer (Fig. 8).

## Materials and methods

### Tissue specimen

Normal cervical epithelial tissue and cervical cancer tissue were obtained from the Third Affiliated Hospital of Guangzhou Medical University. The tissue specimens were immediately stored in liquid nitrogen after collection. Pathological analysis of tissue samples was performed independently by two pathologists. The collected cervical cancer tissues were not treated with radiotherapy or chemotherapy. This study was approved by all subjects and complied with the requirements of the Ethics Committee of Guangzhou Medical University. The tissue specimens are anonymized and strictly comply with the requirements of ethics and laws and regulations.

### Bioinformatics analysis

The cervical cancer RNA-Seq expression data and patient survival data were from UCSC Xena (https://xenabrowser.net/datapages/) and the TCGA database (https://portal.gdc.cancer.gov/). The prognostic analysis was performed by the Kaplan–Meier Plotter (https://kmplot.com/analysis/).

### Cell culture and transfection

Cervical cancer SiHa, HeLa and CaSki cell lines were purchased from Guangzhou Genio Biotechnology Co., Ltd. SiHa, HeLa and CaSki were cultured in DMEM high-glucose medium (GIBCO, USA) supplemented with 10% fetal bovine serum (ExCell Bio, Shanghai) and 1% penicillin-streptomycin (Solarbio, Beijing). The cells were cultured in an incubator with a temperature of 37 °C and a concentration of 5% carbon dioxide. Lipo3000 (Invitrogen, USA) was used for plasmid, ASO oligonucleotide, and siRNA transfection in this study. The SNORD6 overexpression plasmid was constructed using pCDNA3.1(+) vector, and the plasmid carried the G418 resistance tag. After transfection, G418 was used for resistance screening to construct a SNORD6 stable overexpression cell line. The SNORD6 sequence is showed in supplementary file. ASO-SNORD6 and the corresponding control group ASO-NC, si-TP53, si-HPV16E6, si-HPV16E7, si-HPV18E6, si-HPV18E7, and the corresponding control group si-NC were purchased from Guangzhou Ribo Biotechnology Co., Ltd. The sequences are listed in supplementary file.

### CCK-8 assay

The CCK-8 assay was employed to detect cell viability. The adherent cells in logarithmic growth phase were plated in a 96-well cell culture plate (SORFA, China) at 1500 cells per well, and cultured in a cell culture incubator at 37 °C and 5% CO_2_. After the cells adhered, 10 μL of CCK-8 solution (Yisheng, China) was added at 0, 24, 48, and 72 h after the addition of treatment factors (such as transfection). After 2 h of reaction, we performed a microplate reader assay (BioTek, USA). The absorbance values were detected at a wavelength of 450 nm. Finally, we calculated the cell growth rate based on the absorbance value to determine the cell viability.

### Plate cloning assay

Plate cloning assay was applied to establish cell proliferation. The cells in the logarithmic growth phase were plated in a 6-well cell culture plate (SORFA, China) at a density of 500 cells/well, and cultured in a cell culture incubator at 37 °C with 5% CO_2_. The growth of cell clones was observed under a microscope every day and cultured for approximately 2 weeks. After the formation of cell clones (>50 cells) visible to the naked eye, the culture was terminated. The 6-well plate was taken out, washed 2–3 times with PBS buffer, and the cells were fixed with 4% paraformaldehyde solution for 30 min. Washed 2–3 times with PBS buffer and stained with 0.1% crystal violet solution for 10 min. After staining, cells were washed with PBS buffer to remove floating color. After observation with the naked eye, we took pictures and counted the number of the clones formed directly with the naked eye and under a microscope.

### EdU assay

The EdU assay was used to detect DNA synthesis in cells. The cells in the logarithmic growth phase were seeded in a 96-well cell culture plate at a density of 8 * 10^3^ cells/well. After culturing to the normal growth stage, half of the old culture medium was replaced with fresh medium containing 50 μM EdU (Thermo Fisher, USA). The cells were incubated at 37 °C in a cell incubator with 5% CO_2_. After incubation for 2 h, the 96-well plate was taken out, the medium was discarded, and the cells were fixed with 3.7% paraformaldehyde solution at room temperature for 15 min. After the fixation, the cells were washed twice with 3% BSA-containing PBS solution for 5 min each time. The cells were incubated with 5% Triton® X-100 in PBS at room temperature for 20 min, followed by two-fold washing with 3% BSA in PBS for 5 min each time. After configuring Click-iT® Plus reaction (Thermo Fisher, USA) according to the instructions, added 50 μL to each well and incubate at room temperature for 30 min in the dark. Cells were washed once with 3% BSA in PBS and once with PBS. Prepare Hoechst® 33342 solution (final concentration is 5 μg/mL) (Thermo Fisher, USA) according to the instructions, added 50 μL to each well, and incubated at room temperature for 30 min in the dark. Then cells were washed once with PBS solution. After photographing with a fluorescence microscope (Olympus, Japan), the experimental results were analyzed with Image J software.

### Cell cycle assay

Flow PI (propidium iodide) staining experiment was used to detect cellular DNA content. Cells in logarithmic growth phase were seeded in 6-well cell culture plates (2 * 10^5^ cells/well) and cultured at 37 °C under 5% CO_2_ conditions. After cells were adhered, plasmids or ASO oligonucleotides were transfected, and cells were harvested 48 h after transfection. The cells were washed 2–3 times with PBS solution, trypsinized, and centrifuged at 1500 rpm for 5 min. The cells were pelleted and fixed overnight in 70% ice-cold ethanol. The cells were taken out the next day, centrifuged at 1500 rpm for 5 min, the supernatant was removed, washed twice with PBS, and the supernatant was removed. A volume of 500 μL of PI staining solution (BD, USA) was added to each sample, followed by incubation at room temperature for 15 min in the dark, and detection by a flow cytometer (Thermo Fisher Scientific, USA). After the data were collected, FlowJo software (FlowJo, Treestar Inc., Ashland, OR, USA) was utilized to analyze the experimental results and calculate the proportion of the DNA content in each cell cycle.

### Apoptosis assay

Cell apoptosis was assessed by Annexin V-FITC/PI cell staining and flow cytometry. The cells in the logarithmic phase of growth were seeded in a 6-well cell culture plate at a density of 2 * 10^5^ cells/well, and cultured at 37 °C under 5% CO_2_ conditions. After cells adhered, ASO-oligonucleotide or overexpression plasmid treatment was performed, and the culture was terminated 48 h after transfection. Cells were harvested by trypsinization without EDTA, and centrifuged at 2000 rpm for 5 min. The supernatant was discarded, the cells were washed with pre-cooled PBS solution (4 °C), and centrifuged at 2000 rpm for 5 min. After discarding the supernatant, 300 μL of 1 × Binding Buffer (BD, USA) was added to resuspend the cells. Further, 5 μL of Annexin V-FITC (BD, USA) was added, followed by incubation at room temperature for 15 min in the dark and the subsequent addition of 5 μL of PI (BD, USA) staining. Before loading, 200 μL of 1 × Binding Buffer was added. Cell apoptosis was analyzed by flow cytometry (Thermo Fisher Scientific, Waltham, MA, USA), and the experimental results were collected and recorded.

### Transwell experiment

Transwell chambers (Corning, Inc., Corning, NY, USA) with or without Matrigel (Corning, USA) were used to simulate cell migration (HeLa: 2.5 * 10^4^ cells/well; SiHa: 5 * 10^4^ cells/well) and invasion (HeLa: 3.5 * 10^4^ cells/well; SiHa: 8 * 10^4^ cells/well), respectively. Cells in the logarithmic growth phase were plated in the upper chamber coated with or without Matrigel matrix after resuspension in a serum-free medium and then incubated in the lower chamber containing a 10% complete medium. Then, the cells were cultured in a cell incubator for 48 h. The medium in upper and lower chambers were discarded, and the cells were washed 2–3 times with PBS. After fixing the cells with 4% paraformaldehyde at room temperature for 30 min, the cells were washed 2–3 times with PBS solution. Further, staining with crystal violet solution was performed at room temperature for 10 min. PBS solution was used to wash off the floating color, and the cells in the upper chamber that did not pass through the bottom membrane of the chamber were wiped off with a cotton swab. The cells that passed through the bottom membrane of the chamber were observed under a microscope and photographed. The images of the cells were analyzed with Image J software.

### In vivo tumorigenesis

In this study, female BALB/c nude mice (4–6 weeks, 16–18 g) were used for in vivo tumorigenesis. 1 * 10^7^ HeLa was resuspended in 100 μl of serum-free medium and 100 μl of Matrigel mixture (Corning, USA), and the cell suspension was inoculated in the axilla of nude mice. When the tumor volume reached 100 mm^3^, the nude mice were equally divided into 2 groups with 4 mice in each group. One group was injected with ASO-NC; the other group was injected with ASO-SNORD6 (RiboBio, Guangzhou). Administer once every 3 days, with a dose of 10 nmol per animal, for a total of 3 weeks. The formula for calculating tumor volume is (length diameter × short diameter ^2^)/2.

### Quantitative real‑time PCR (qRT‑PCR)

After collecting the tissues or cells, we added 1 mL of TRIzol solution (TaKaRa, Japan) and subjected them to complete lysis for 5–10 min. Further, we added 200 μL of chloroform, followed by vigorously shaking, incubation at room temperature for 5 min, and centrifugation at 12,000 rpm for 15 min. Next, the supernatant was collected, and an equal volume of isopropanol was added to dissolve it at room temperature for 10 min, followed by centrifugation at 12,000 rpm for 15 min. The precipitate was then collected, washed with 75% ethanol, and centrifuged at 7500 rpm for 10 min. We discarded the supernatant, dried the residual liquid, and added 10–30 μL of DEPC water to dissolve the RNA. After the RNA concentration test, 1 μg specimens were reverse-transcribed using Hifair® III 1st Strand cDNA Synthesis SuperMix for qPCR (gDNA digester plus) (Yeasen Biotechnology, Shanghai, China). The cDNA obtained by reverse transcription was detected by real-time quantitative PCR using Hieff® qPCR SYBR Green Master Mix (Yeasen Biotechnology, Shanghai). The utilized primer sequences are listed in supplementary file. After the amplification, with U6/GAPDH as the internal reference, the 2^-ΔΔct^ method was applied to calculate the expression level of the target gene.

### Western blotting

After the cells were collected, they were fully lysed using RIPA lysis buffer (BestBio, Shanghai). Then, the supernatant was collected, and the protein concentration was detected by the BCA method; each group of proteins was prepared as a mixture of equal concentrations and equal volumes. Further, 30 μg of total protein was subjected to sodium dodecyl sulfate-polyacrylamide gel electrophoresis (SDS-PAGE). The protein on the gel was transferred to a PVDF membrane. The membranes were next blocked with 5% skim milk for 2 h and incubated with a primary antibody at 4 °C overnight. The next day, the membrane was washed three times with TBST for 10 min, and the corresponding secondary antibody solution was added according to the properties of the primary antibody, and incubated at room temperature for 1.5 h. Washing with TBST three times, for 10 min each time was applied. Then, ECL luminescence was performed using a chemiluminometer (BIO-RAD, USA), and the gray value of protein bands was analyzed with Image J software. The antibodies and dilution ratios used were: HPV16 E6/18 E6 (C1P5) (1:200; Santa Cruz Biotechnology, sc-460); HPV16 E7 (ED17) (1:500; Santa Cruz Biotechnology, sc-6981); HPV18 E7 (F-7) (1:500; Santa Cruz Biotechnology, sc-365035); P53 Monoclonal antibody (1:5000; Proteintech, 60283-2-Ig); P21 Polyclonal antibody (1:1000; Proteintech, 10355- 1-AP); E6AP/UBE3A Polyclonal antibody (1:1000; Proteintech, 10344-1-AP); BAX Polyclonal antibody (1: 5000; Proteintech, 50599-2-Ig); HRP-conjugated Affinipure GoatAnti-Mouse IgG (H + L) (1:5000; Proteintech, SA00001-1); and HRP-conjugated Affinipure GoatAnti-Rabbit IgG (H + L) (1:8000; Proteintech, SA00001-2).

### RNA-binding protein immunoprecipitation assay (RIP)

Cells (4 * 10^7^) were collected from each group, fully lysed with RIP lysate, and centrifuged at 4 °C, 12,000 rpm for 10 min. A volume of 20 μL of the cell lysate supernatant was taken, which served as the input group. We then added the antibody and IgG control that have been pre-conjugated with Protein A/G magnetic beads to the remaining part, and incubated the samples at 4 °C under constant temperature stirring for 12–16 h. The magnetic beads were washed six times with RIP wash buffer on the second day. After the magnetic beads were treated with Proteinase K, they were resuspended in TRIzol solution, followed by RNA extraction, reverse transcription, and the q-PCR steps specified above.

### Co-immunoprecipitation (Co-IP)

We collected 1 * 10^7^ cells in each group and subjected them to complete lysis with immunoprecipitation buffer supplemented with protease inhibitors. Then, the lysate was centrifuged at 4 °C, 12,000 rpm for 10 min, and the supernatant was collected for later use. Antibodies and IgG control were added to the supernatant, and the mixture was inverted and mixed overnight at 4 °C. On the second day, we added 20 μL Protein A/G magnetic beads, inverted and mixed the samples at 4 °C for 2–4 h. After washing the beads five times with washing buffer, we discarded the washing buffer. Then, we resuspended the magnetic beads with 20–40 μL of 2X SDS loading buffer, boiled the samples at 95–100 °C for 5–10 min, and performed a Western blot analysis.

### Ubiquitination assay

After the cells transfected with the SNORD6 overexpression plasmid and treated with 20 μM MG132 for 6 h, the cells were harvested and lysed with cell lysate. The cell lysate was centrifuged, and the supernatant was taken and incubated with an anti-p53 antibody at 4 °C overnight. The subsequent steps are the same as described above in the Co-IP sub-section. The changes in the expression level of ubiquitin were detected by Western blot analysis.

### Fluorescence in situ hybridization (FISH)

This experiment was carried out according to the instructions of the RiboTM Fluorescent In Situ Hybridization Kit (RiboBio, Guangzhou) utilized for our experiments. The cell slides were placed at the bottom of a six-well cell culture plate, and cells were seeded at a density of 1 * 10^5^/well. When the cell confluency reached 60–70%, cells were fixed with 4% paraformaldehyde at room temperature for 10 min. Then, the cells were washed three times with 1× PBS solution for 5 min each. Next, we permeabilize the cells with permeabilization solution (PBS containing 0.5% Triton X-100) at 4 °C for 5 min, followed by washing three times with 1× PBS solution for 5 min each. The pre-hybridization solution was blocked at 37 °C for 30 min, and the probe hybridization solution was added at 37 °C for overnight hybridization. The next day, the cells were washed with hybridization washing solution and stained with DAPI staining solution for 10 min. After removing the cell slides, pictures were taken with a confocal microscope (Nikon, Japan). The probe sequences were provided by RiboBio (Guangzhou).

### Immunofluorescence (IF)

The cell slides were placed in a six-well cell culture plate, and cells in the logarithmic growth phase were selected and plated on cell slides. When the cells grew to 80–90% confluence, they were fixed with 4% paraformaldehyde at room temperature for 30 min. Cells were permeabilized with PBS containing 0.5% Triton X-100 for 15 min at room temperature and washed three times with PBS solution for 5 min each. Then, incubation with the primary antibody was performed overnight at 4 °C. The next day, the cells were washed three times with PBS solution for 5 min each, and incubated with fluorescent secondary antibodies for 1.5 h at room temperature in the dark. After washing the cells three times with PBS solution, the nuclei were stained with DAPI solution for 10 min. The cells were washed three times with PBS solution, mounted on slides, and photographed under a confocal microscope (Nikon, Japan). The antibodies used were: HPV16 E6/18 E6 (C1P5) (Santa Cruz Biotechnology, sc-460); P53 Monoclonal antibody (Proteintech, 60283-2-Ig); E6AP/UBE3A Polyclonal antibody (Proteintech, 10344-1-AP); Fluorescein (FITC)–conjugated Affinipure Goat Anti-Rabbit IgG(H + L) (Proteintech, SA00003-1); Fluorescein (FITC)–conjugated Affinipure Goat Anti-Rabbit IgG(H + L) (Proteintech, SA00003-2).

### Statistical analysis

All aforementioned experiments were repeated three times independently. The obtained data were expressed as mean ± SD or median (quartile). Data were statistically analyzed using SPSS 25.0 and GraphPad Prism 8, the variables of two groups was performed by two-tailed Student’s *t* test, the variables of three or more groups was performed by one-way analysis of variance (ANOVA). *P* < 0.05 was considered to indicate a statistically significant difference.

## Supplementary information

supplementary table.

supplementary file

supplementary figure legends

supplementary Figure 1

supplementary Figure 2

supplementary Figure 3

Original full length western blots

## Data Availability

All data are available in the main text or the supplementary materials.
