# Supplementary material for: SNORD6 promotes cervical cancer progression by accelerating E6-mediated p53 degradation
Source: Cell Death Discov. 2023 Jun 27;9:192. doi: 10.1038/s41420-023-01488-w (PMC10300194; doi:10.1038/s41420-023-01488-w)
Supplement: Supplementary file 1 — supplementary table. [file 41420_2023_1488_MOESM1_ESM.doc]

**Supplementary Table 1:** **SNORD6** expression in normal cervical epithelial tissues and cervical cancer tissues

| **Groups** | **N** | **SNORD6/ U6** | ***P* value** |
| --- | --- | --- | --- |
|
| Normal cervical epithelial tissues | 29 | 3.40146E-05 ±2.16721E-05 |  |
| Cervical cancer tissues | 97 | 8.20746E-05 ±5.96401E-05 | ***<0.0001*** |

Bold and Italics means P < 0.05.

**Supplementary Table 2:** Correlation of **SNORD6** expression with different clinicopathological features of cervical cancer

| **Clinicopathological**  **features** | **N** | **SNORD6/ U6** | ***P* value** |
| --- | --- | --- | --- |
|
| **FIGO stages** |  |  | ***0.0360*** |
| I | 62 | 7.23331E-05±4.58729E-05 |  |
| II-IV | 35 | 0.000101374±7.53076E-05 |  |
| **Pathology classification** |  |  | ***0.0315*** |
| Well+ Mod | 40 | 6.86434E-05±4.98138E-05 |  |
| Poor | 57 | 9.15E-05±6.44221E-05 |  |
| **Age** |  |  | 0.1168 |
| ≤ 48 | 50 | 7.2825E-05±5.30778E-05 |  |
| > 48 | 47 | 9.19146E-05±6.50405E-05 |  |
| **The pathology types** |  |  | 0.9900 |
| Squamous carcinoma | 77 | 8.21248E-05±5.94949E-05 |  |
| The other pathology types | 20 | 8.18813E-05±6.17518E-05 |  |
| Bold and Italics means P < 0.05. | | | |
