# Supplementary material for: SNORD6 promotes cervical cancer progression by accelerating E6-mediated p53 degradation
Source: Cell Death Discov. 2023 Jun 27;9:192. doi: 10.1038/s41420-023-01488-w (PMC10300194; doi:10.1038/s41420-023-01488-w)
Supplement: Supplementary file 2 — supplementary file [file 41420_2023_1488_MOESM2_ESM.docx]

**Supplementary file**

**1. SNORD6 sequence:** GATGTTATGATGATGGGCGAAATGTTCAACTGCTCTGAAGGGGCTGAATGAAAATGGCCTTTCTGAACATC.

**2. The sequences of ASO-SNORD6/ si-TP53/ si-HPV16E6/ si-HPV16E7/ si-HPV18E6/ si-HPV18E7:**

| ASO-SNORD6 | ATGATGATGGGCGAAATGTT |
| --- | --- |
| si-TP53 | GTACCACCATCCACTACAA |
| si-HPV16E6 | GTATGGAACAACATTAGAA |
| si-HPV16E7 | GCATGGAGATACACCTACA |
| si-HPV18E6 | CAGACTCTGTGTATGGAGA |
| si-HPV18E7 | CGATGAAATAGATGGAGTT |

Both ASO-NC and si-NC sequences were provided by Guangzhou Ribo Biotechnology Co., Ltd.

**3. The primer sequences:**

| SNORD6 | F: 5' GATGGGCGAAATGTTCAACTGCTCT 3' |
| --- | --- |
|  | R: 5' CCATTTTCATTCAGCC 3' |
| HPV16E6 | F: 5' GCAAGCAACAGTTACTGAGACGT 3' |
|  | R: 5' GCAACAAGACATACATCGACCGG 3' |
| HPV16E7 | F: 5' ATAATATAAGGGGTCGGTGG 3' |
|  | R: 5' CATTTTCGTTCTCGTCATCTG 3' |
| HPV18E6 | F: 5' TGGTGTATAGAGACAGTATACCCC 3' |
|  | R: 5' GCCTCTATAGTGCCCAGCTATGT' 3' |
| HPV18E7 | F: 5' TACCCAAGCTTCATGGACCTAAGGCAACATTG 3' |
|  | R: 5' TACGCGGATCCTTACTGCTGGGATGCACACC' 3' |
| TP53 | F: 5′ CAGCACATGACGGAGGTTGT 3′ |
|  | R: 5′ TCATCCAAATACTCCACACGC 3′ |
| UBE3A | F: 5' CTCAGCTTACCTTGAGAACTCG 3' |
|  | R: 5' TTCTAGCGCCTTTCTTGTTCAT 3' |
| U6 | F: 5' CTCGCTTCGGCAGCACA 3' |
|  | R: 5' AACGCTTCACGAATTTGCGT 3' |
| GAPDH | F: 5'-CCCATCACCATCTTCCAGGAG-3' |
|  | R: 5'-GTTGTCATGGATGACCTTGGC-3' |
| CDKN1A | F: 5'-TTCTACCACTCCAAACGCCG-3' |
|  | R: 5'-GGCAGAAGATGTAGAGCGGG-3' |
| BAX | F: 5'-AGAGGTCTTTTTCCGAGTGGC-3' |
|  | R: 5'-AGTAGAAAAGGGCGACAACCC-3' |
