# Supplementary material for: SNORD6 promotes cervical cancer progression by accelerating E6-mediated p53 degradation
Source: Cell Death Discov. 2023 Jun 27;9:192. doi: 10.1038/s41420-023-01488-w (PMC10300194; doi:10.1038/s41420-023-01488-w)
Supplement: Supplementary file 3 — supplementary figure legends [file 41420_2023_1488_MOESM3_ESM.docx]

**SUPPLEMENTARY FIGURE LEGENDS**

**Supplementary Fig.1 The role of SNORD6 in CaSki**

A. The expression of SNORD6 in cervical cancer cell lines SiHa, HeLa and CaSki was detected by q-PCR.

B. Q-PCR showed the transfection efficiency after transfection of SNORD6 overexpression plasmid in CaSki.

C-E. Overexpression of SNORD6 in CaSki resulted in increased cell growth (C), increased colony formation (D) and decreased apoptosis (E).

All experiments were repeated three times independently. The above data are expressed as mean ± SD. Student’s t test. *, p<0.05, **, p<0.01, ****, p<0.0001.

**Supplementary Fig.2 SNORD6 affects p53 expression in CaSki**

A. Overexpression of SNORD6 in CaSki resulted in increased E6 protein expression and decreased p53 protein expression, while SNORD6 did not affected E6AP expression.

B. Q-PCR showed after SNORD6 overexpression, the mRNA expression of CDKN1A and BAX was decreased in CaSki.

C. Western blotting results suggested overexpression of SNORD6 in CaSki caused decreased protein expression of p21and BAX.

All experiments were repeated three times independently. The above data are expressed as mean ± SD. Student’s t test. *, p<0.05, ***, p<0.001.

**Supplementary Fig.3 TP53 overexpression can enhance the ASO-SNORD6 effects in HeLa**

A. After TP53 overexpression plasmid transfection, the TP53 expression was significantly increased.

B-D. The slowing down of cell proliferation (B), increased apoptosis (C), and increased expression of p53 and its downstream target proteins (D) caused by SNORD6 knockdown can be further enhanced by overexpression of TP53.

All experiments were repeated three times independently. The above data are expressed as mean ± SD. One-way ANOVA. *, p<0.05, **, p<0.01, ****, p<0.0001.
