# Supplementary figures and images for: SNORD6 promotes cervical cancer progression by accelerating E6-mediated p53 degradation
Source: Cell Death Discov. 2023 Jun 27;9:192. doi: 10.1038/s41420-023-01488-w (PMC10300194; doi:10.1038/s41420-023-01488-w)

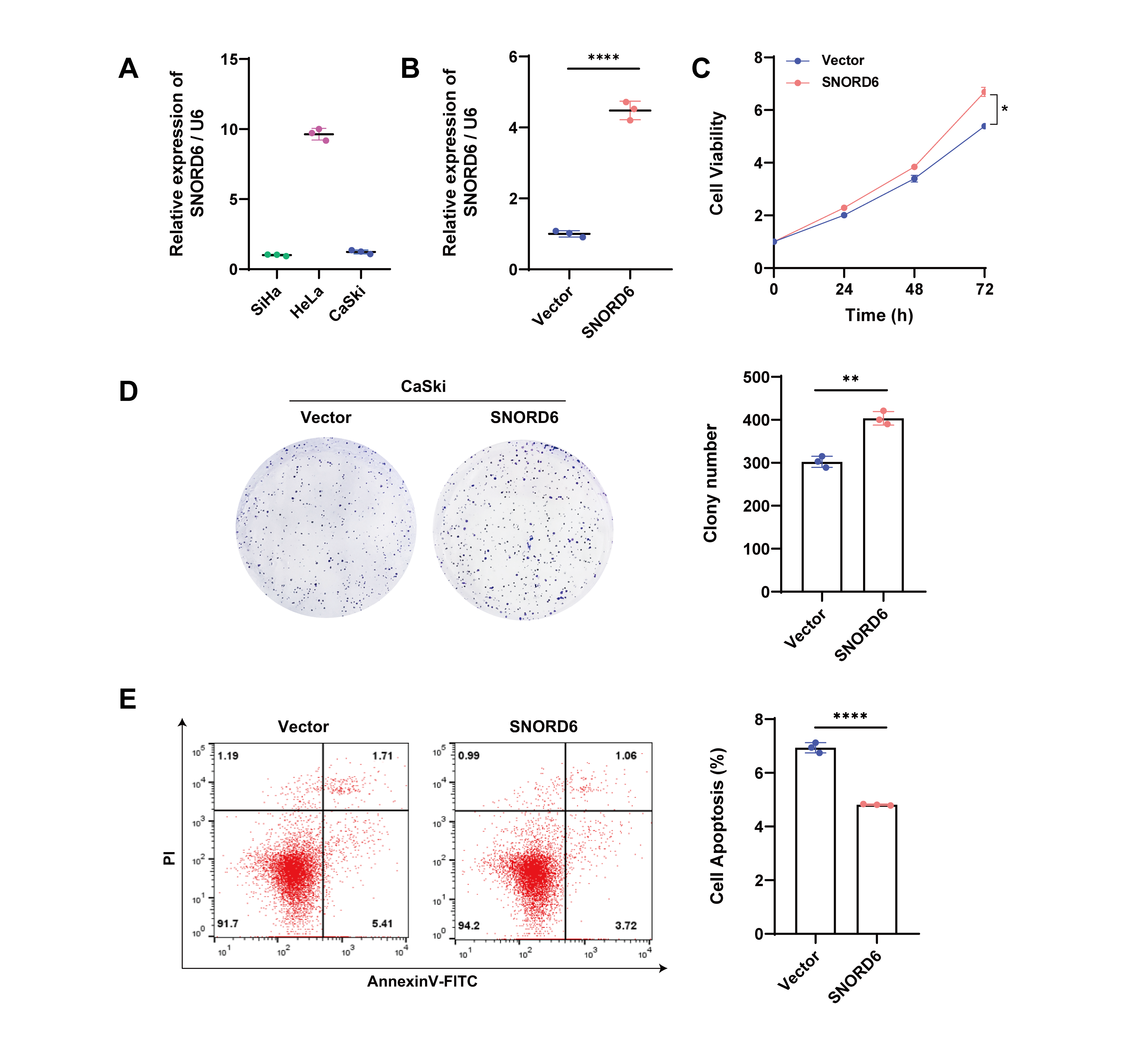

Supplement: Supplementary file 4 — supplementary Figure 1 [file 41420_2023_1488_MOESM4_ESM.png]

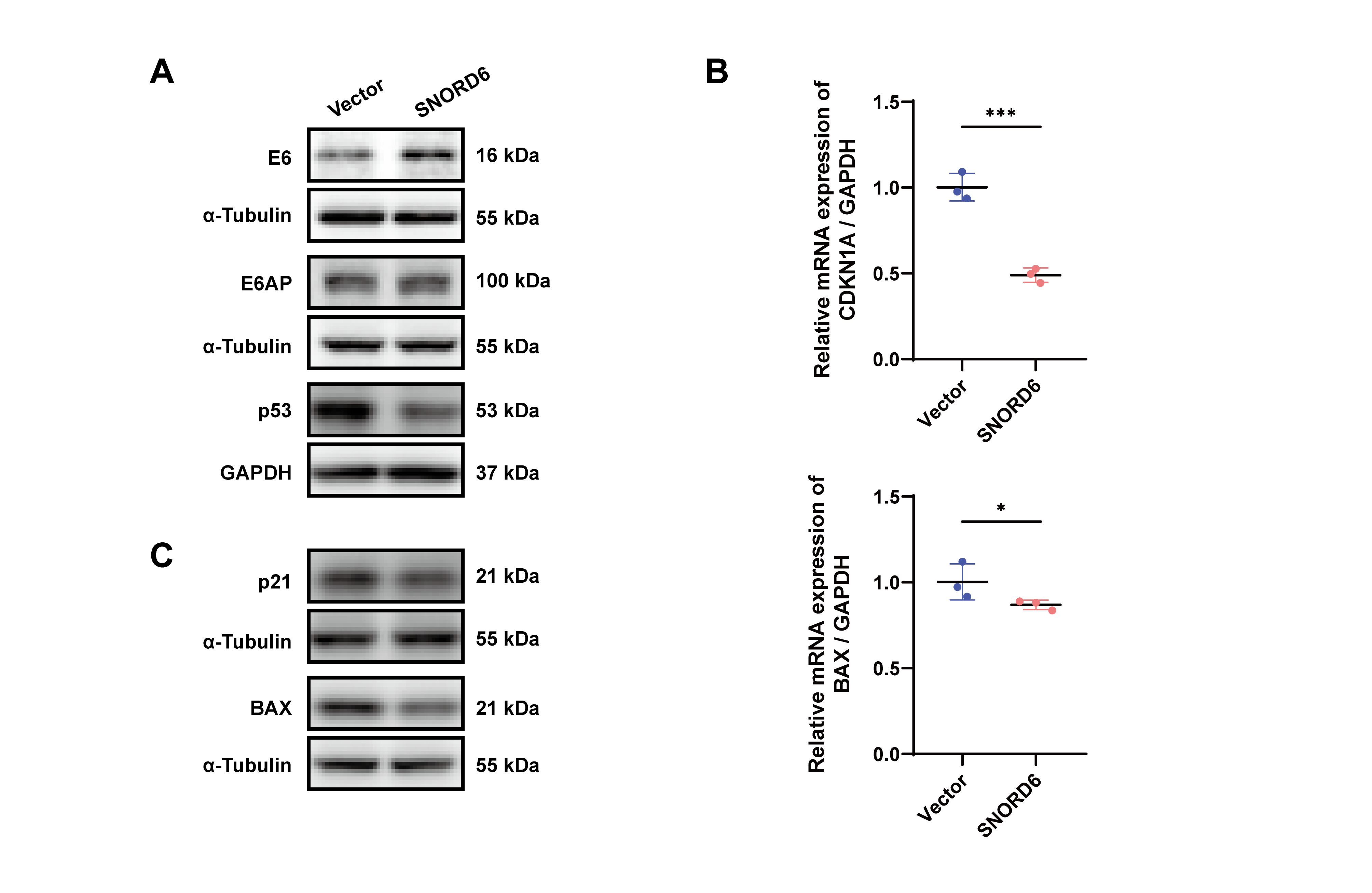

Supplement: Supplementary file 5 — supplementary Figure 2 [file 41420_2023_1488_MOESM5_ESM.png]

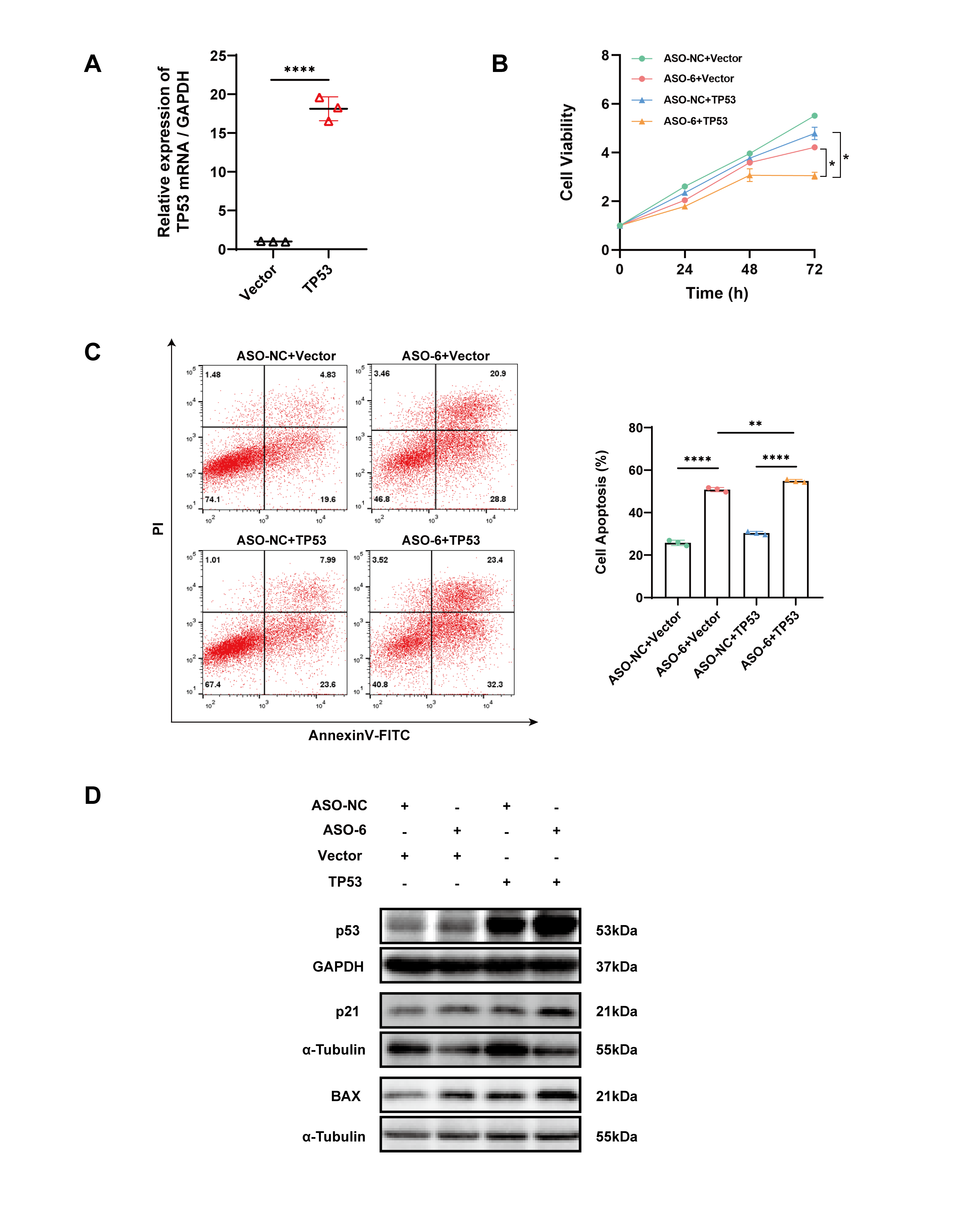

Supplement: Supplementary file 6 — supplementary Figure 3 [file 41420_2023_1488_MOESM6_ESM.png]
