# Supplementary material for: SNORD6 promotes cervical cancer progression by accelerating E6-mediated p53 degradation
Source: Cell Death Discov. 2023 Jun 27;9:192. doi: 10.1038/s41420-023-01488-w (PMC10300194; doi:10.1038/s41420-023-01488-w)

Original full length western blots

Figure 4

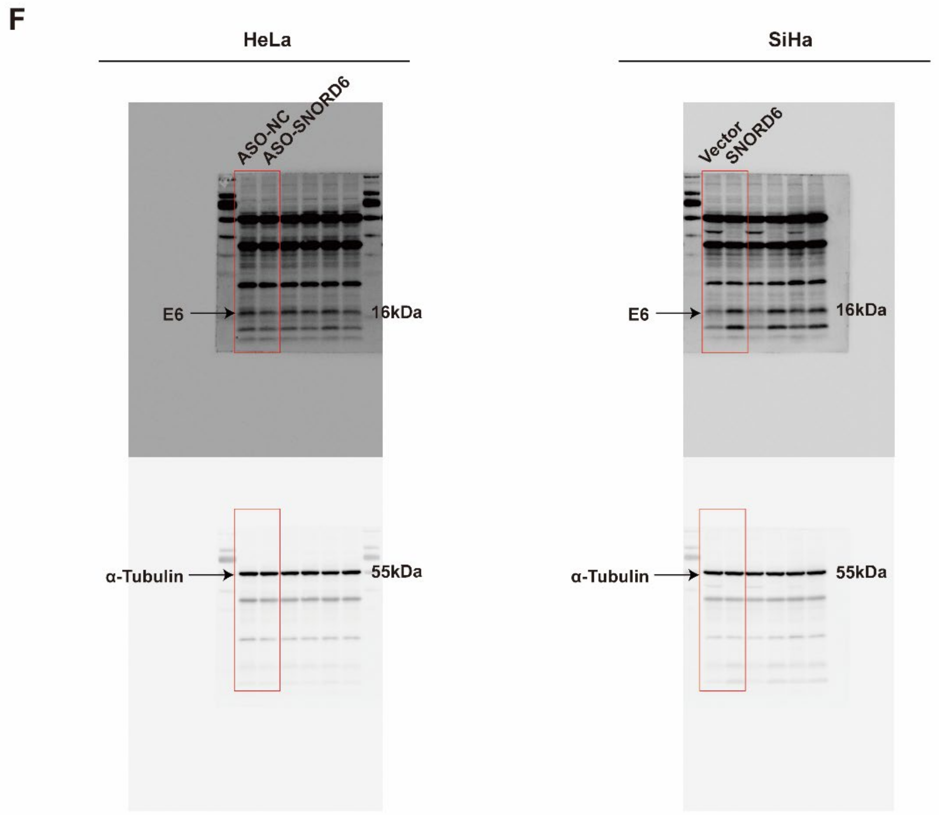

Figure 5

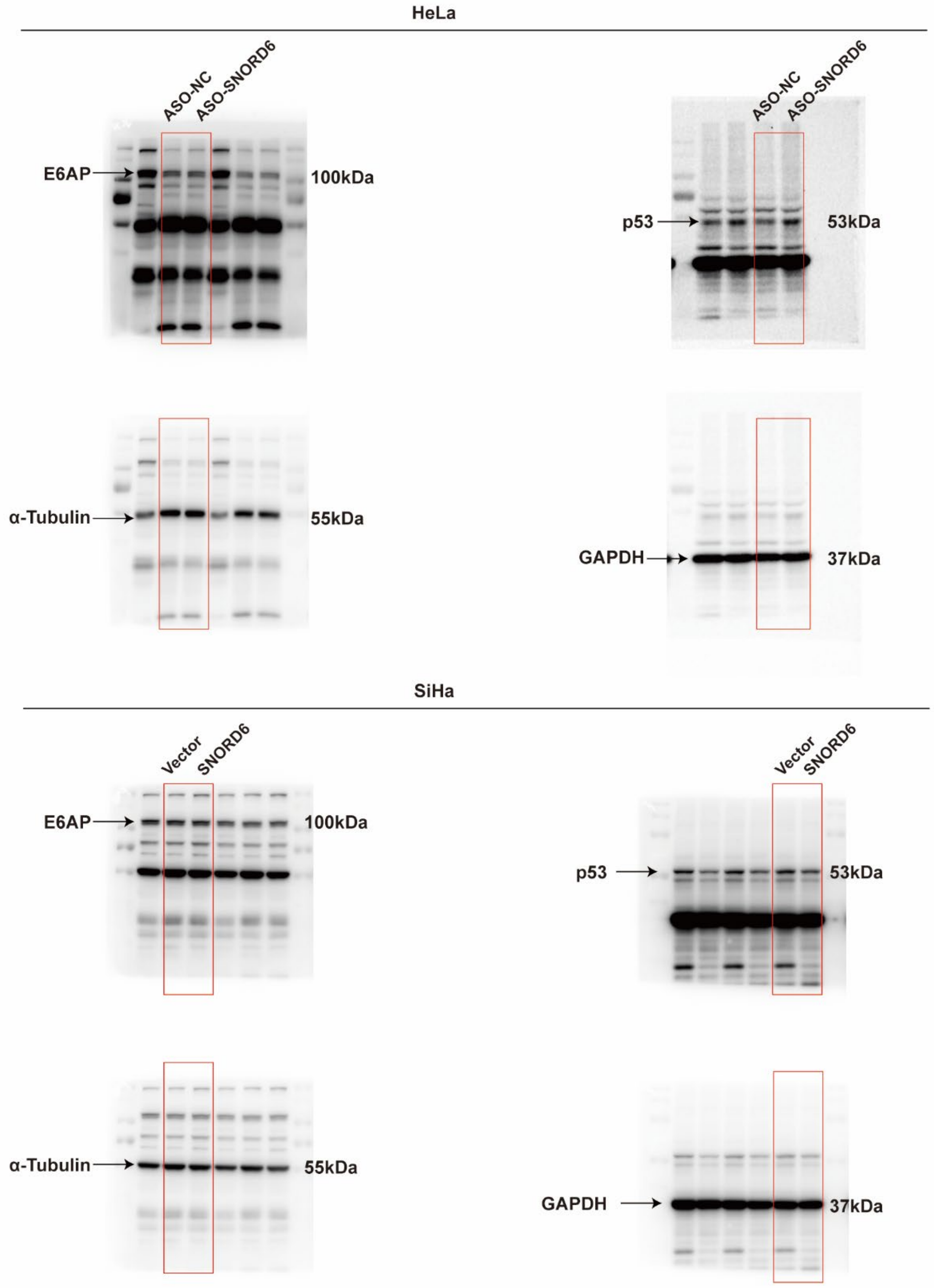
HeLa
B
ASO-NC
ASO-SNORD6
E6AP
100kDa
p53
53kDa
α-Tubulin
55kDa
GAPDH
37kDa
SiHa
Vector
SNORD6
E6AP
100kDa
p53
53kDa
α-Tubulin
55kDa
GAPDH
37kDa

Figure 5

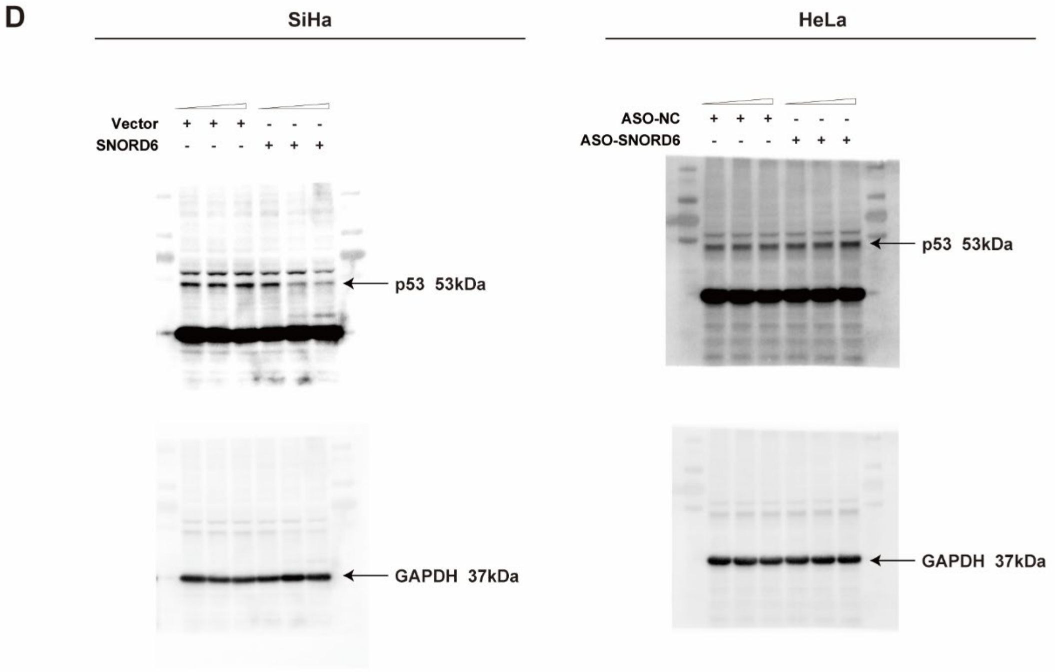
D
SiHa
HeLa
Vector + + + - - -
SNORD6 - - - + + +
p53 53kDa
GAPDH 37kDa
ASO-NC + + + - - -
ASO-SNORD6 - - - + + +
p53 53kDa
GAPDH 37kDa

Figure 5

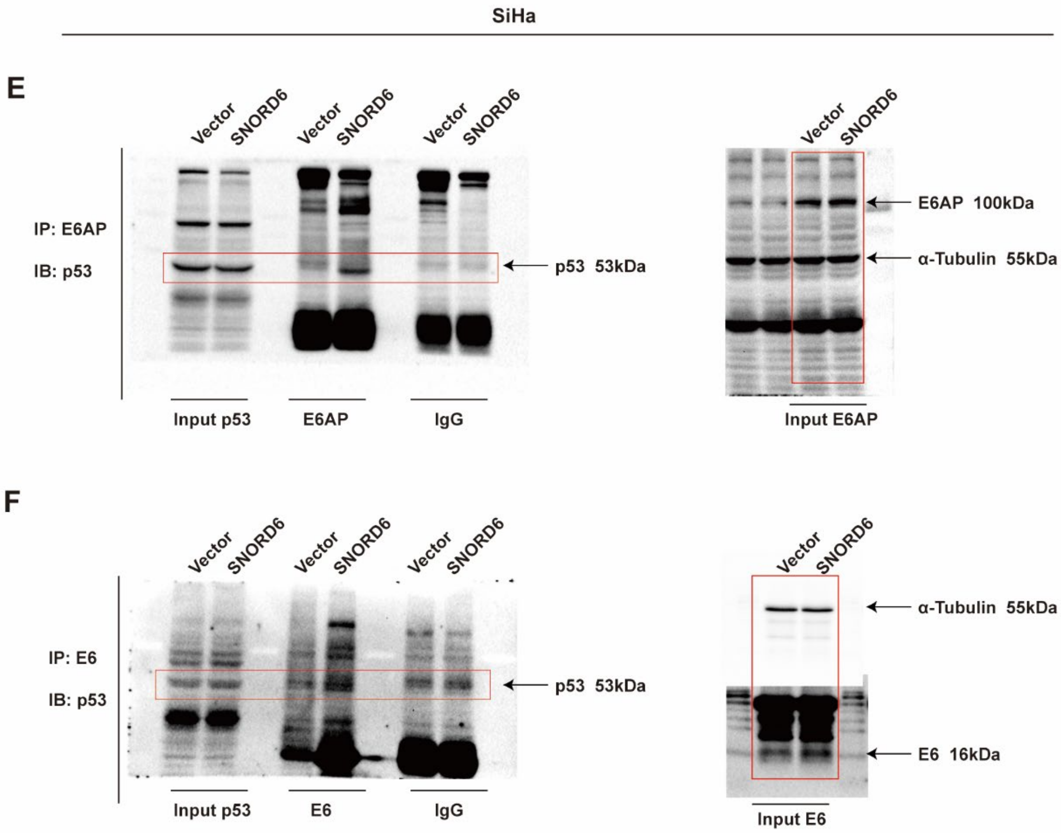
SiHa
E
Vector
SNORD6
IP: E6AP
IB: p53
p53 53kDa
Input p53
E6AP
IgG
E6AP 100kDa
α-Tubulin 55kDa
Input E6AP
F
IP: E6
IB: p53
Input p53
E6
E6 16kDa
Input E6

Figure 6

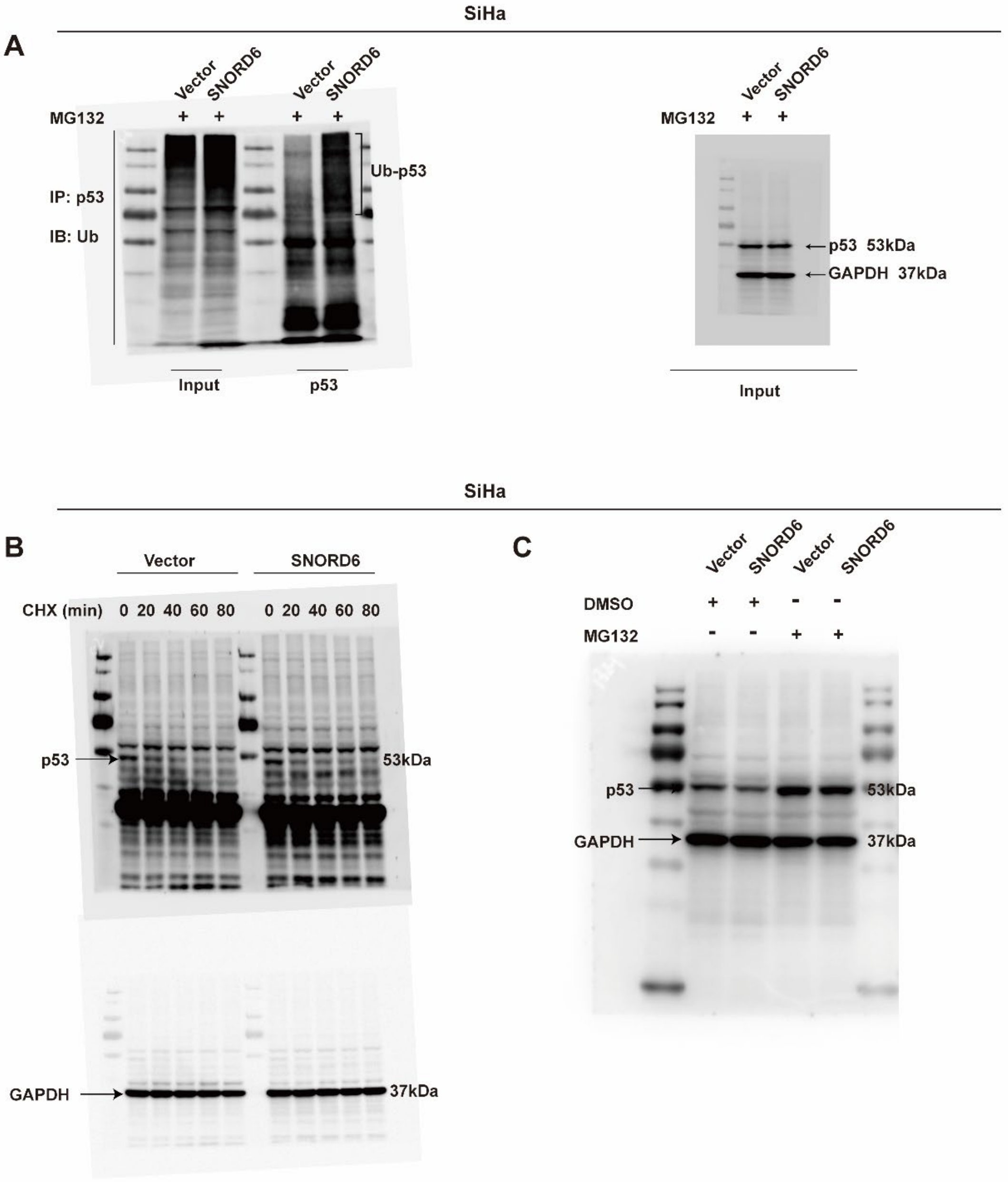
SiHa
A
Vector
SNORD6
Vector
SNORD6
MG132
+
+
+
+
IP: p53
IB: Ub
Ub-p53
Input
p53
Vector
SNORD6
MG132
+
+
p53 53kDa
GAPDH 37kDa
Input
SiHa
B
Vector
SNORD6
CHX (min)
0 20 40 60 80
0 20 40 60 80
p53
53kDa
GAPDH
37kDa
C
Vector
SNORD6
Vector
SNORD6
DMSO
+
+
-
-
MG132
-
-
+
+
p53
53kDa
GAPDH
37kDa

Figure 6

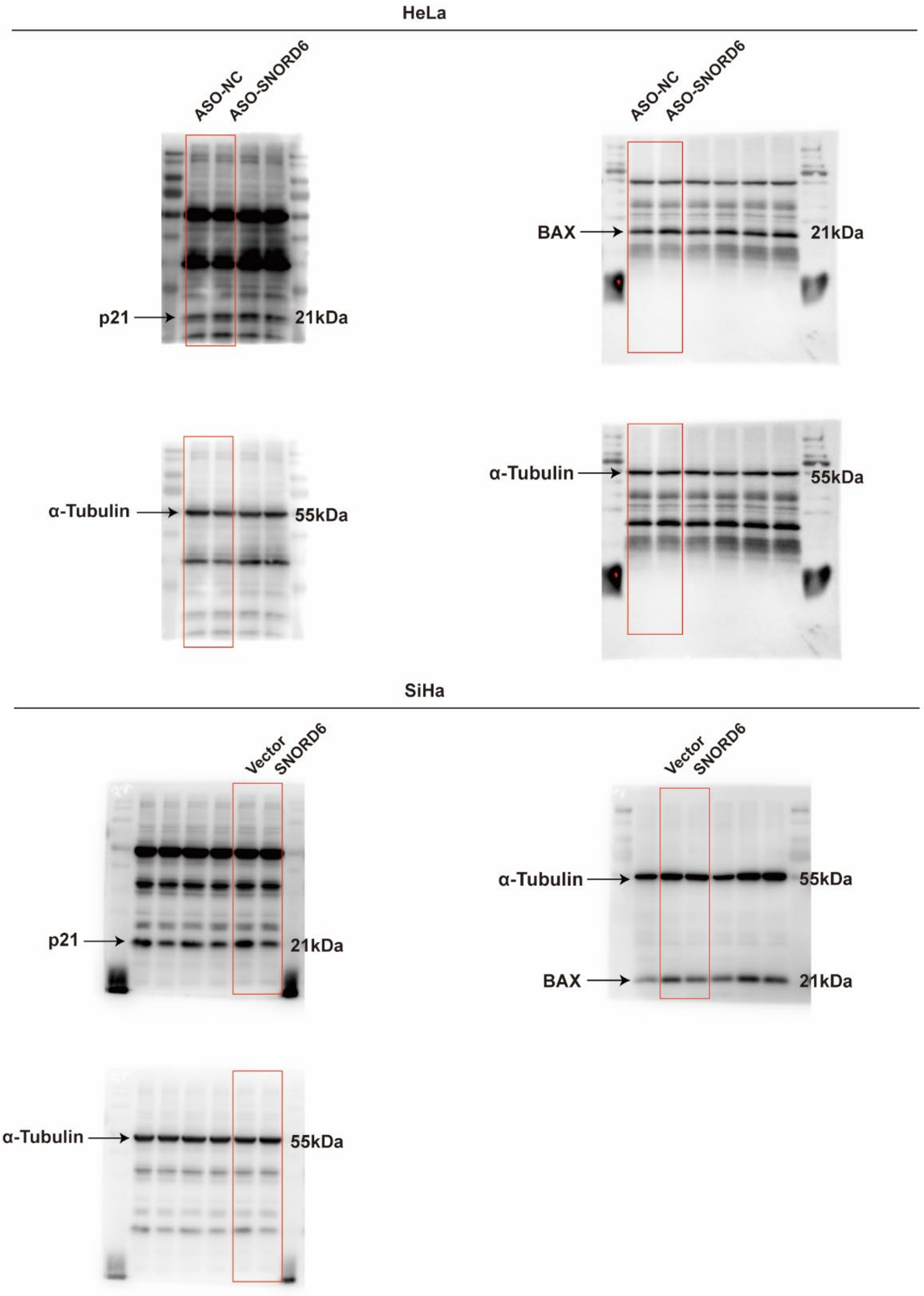
HeLa
E
ASO-NC
ASO-SNORD6
p21
21kDa
α-Tubulin
55kDa
BAX
21kDa
α-Tubulin
55kDa
SiHa
Vector
SNORD6
p21
21kDa
α-Tubulin
55kDa
α-Tubulin
55kDa
BAX
21kDa

Figure 7

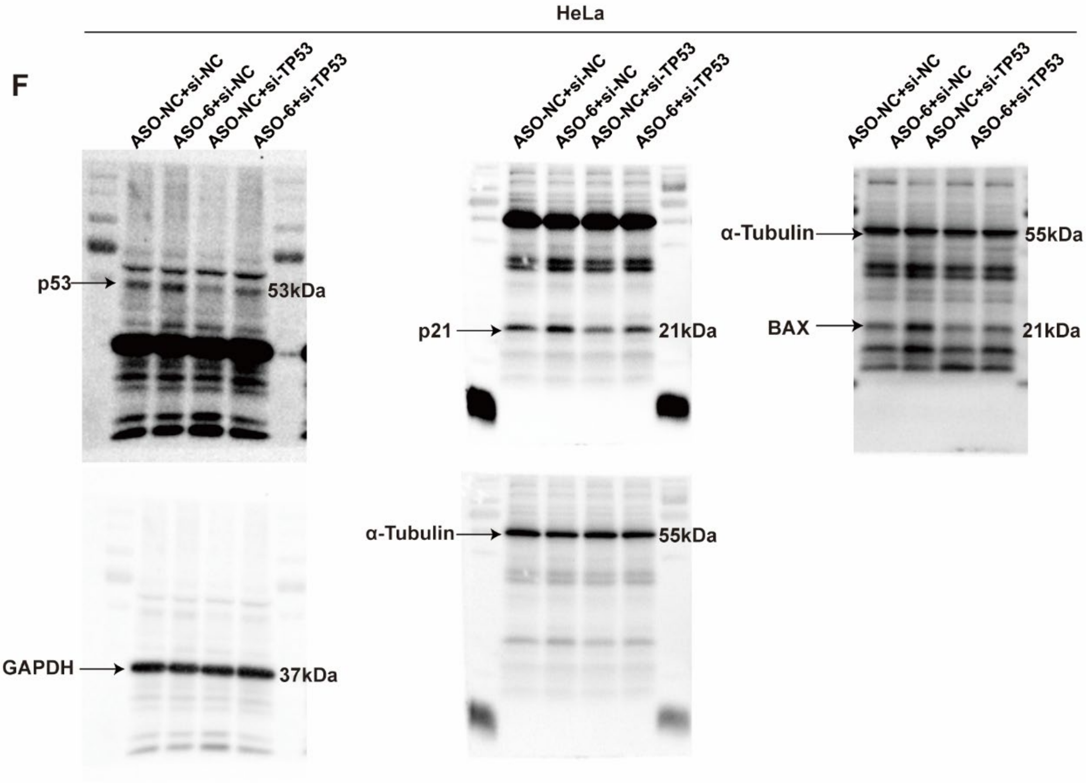

HeLa
F
ASO-NC+si-NC
ASO-6+si-NC
ASO-NC+si-TP53
ASO-6+si-TP53
p53
53kDa
GAPDH
37kDa
p21
21kDa
α-Tubulin
55kDa
α-Tubulin
55kDa
BAX
21kDa

Supplementary Figure 2

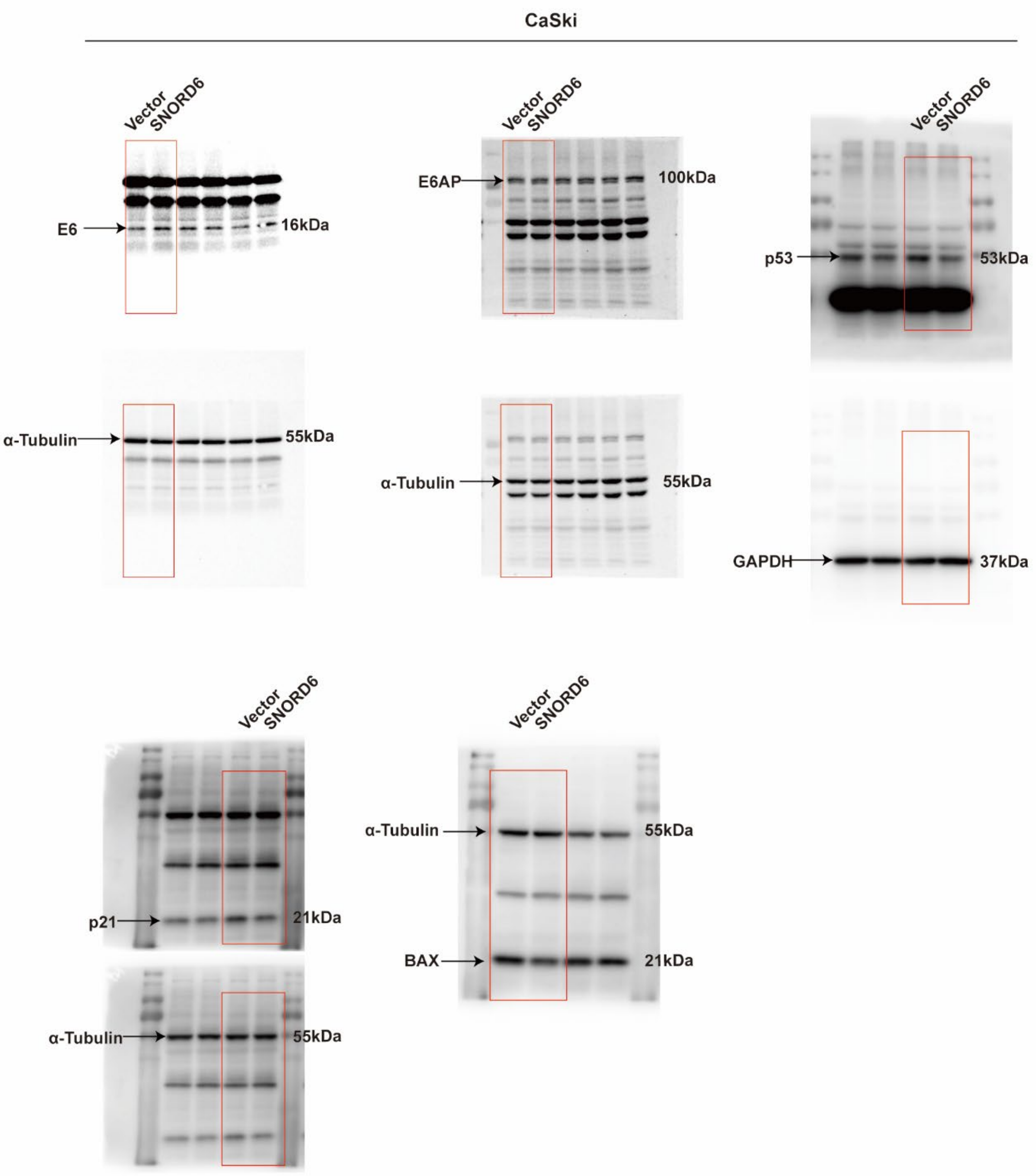

Supplementary Figure 3

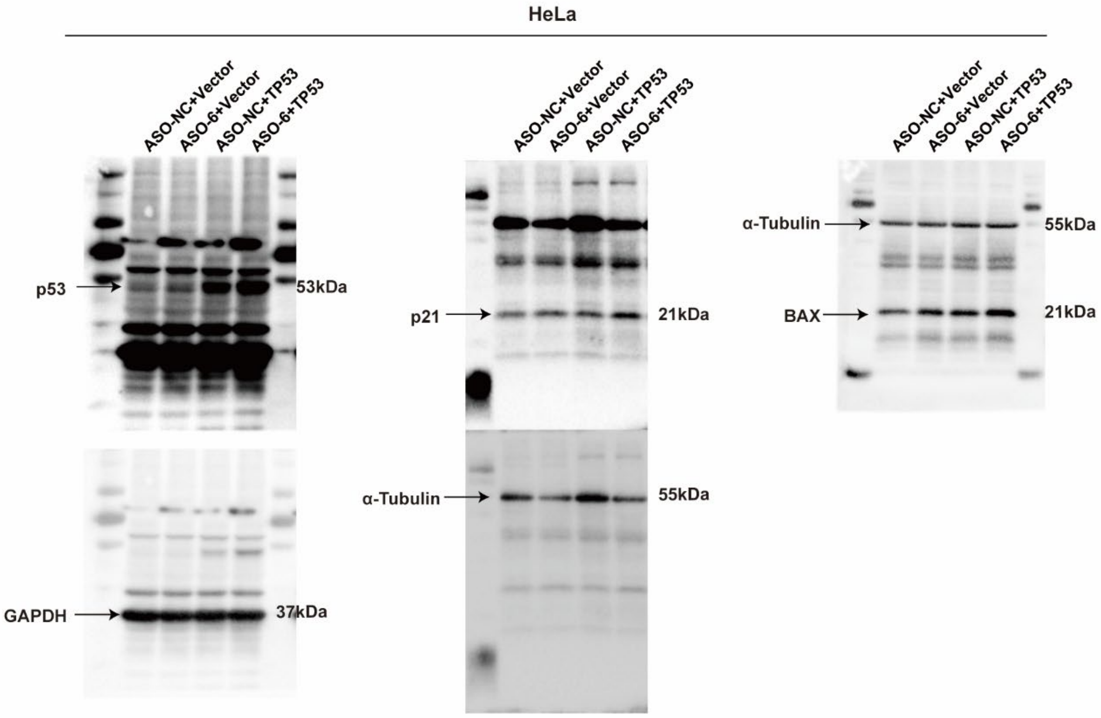

Supplement: Supplementary file 7 — Original full length western blots [file 41420_2023_1488_MOESM7_ESM.pdf]
